# PLAT: An Automated Fault and Behavioural Anomaly Detection Tool for PLC Controlled Manufacturing Systems

**DOI:** 10.1155/2016/1652475

**Published:** 2016-11-07

**Authors:** Arup Ghosh, Shiming Qin, Jooyeoun Lee, Gi-Nam Wang

**Affiliations:** ^1^Unified Digital Manufacturing Laboratory, Department of Industrial Engineering, Ajou University, Suwon 443-749, Republic of Korea; ^2^Department of Industrial Engineering, Ajou University, Suwon 443-749, Republic of Korea

## Abstract

Operational faults and behavioural anomalies associated with PLC control processes take place often in a manufacturing system. Real time identification of these operational faults and behavioural anomalies is necessary in the manufacturing industry. In this paper, we present an automated tool, called PLC Log-Data Analysis Tool (PLAT) that can detect them by using log-data records of the PLC signals. PLAT automatically creates a nominal model of the PLC control process and employs a novel hash table based indexing and searching scheme to satisfy those purposes. Our experiments show that PLAT is significantly fast, provides real time identification of operational faults and behavioural anomalies, and can execute within a small memory footprint. In addition, PLAT can easily handle a large manufacturing system with a reasonable computing configuration and can be installed in parallel to the data logging system to identify operational faults and behavioural anomalies effectively.

## 1. Introduction

Currently, most of the manufacturing systems are controlled by PLC because of its adaptability, modularity, user-friendliness, and low cost [1–4]. Operational faults associated with PLC control process occur most often (about 70%) among all kinds of faults [3, 5]. It is very difficult to identify faults and their detailed effects in a PLC controlled manufacturing system because PLC has a very inflexible programming system, and PLC device or its control system does not contain any built-in module for this task [4] (in this work, the term “fault” refers to the operational fault). Significant damage to the system can result if the faults propagate though the system. Moreover, it becomes harder to distinguish the root cause of the fault once it propagates through the system and, hence, real time detection of a fault is necessary in the manufacturing industry in order to save them from great loss of revenue. Usually, maintenance and operation engineers find it difficult to repair the fault sources because of the lack of knowledge about control logics and the fault's characteristics. These issues can be addressed by providing engineers an easy understandable representation of the control characteristics and knowledge about the fault. The detection of behavioural anomalies associated with control process is another important requirement of the manufacturing industry and receives increasing attention from researchers. This is mainly because it is possible to get a clear indication of the fault prior to its occurrence from the deviation of the control process behaviour. Moreover, behavioural anomaly detection can also help operators to identify several other issues such as device performance degradation and increment in production of low quality items. There is an increasing need for an automated tool that can solve the above-mentioned purposes that we have addressed in this work.

In past three decades, a lot of research works on fault detection in PLC controlled manufacturing systems have been done. In past work [4], a hamming distance based signal pattern matching mechanism for fault detection was proposed by Qin and Wang. In [6], an approach was taken that automatically generates the PLC code for process observation and fault detection. In related article [3], a method was proposed that automatically generates a knowledge base from the PLC program and pneumatic and hydraulic circuit diagram; and using that knowledge base performs fault diagnosis. Two complementary models, that is, logical and sequential diagnosis model, were proposed by Hu et al. [2] to satisfy the same purpose (a similar approach was proposed in [7]). In another paper [5], a hierarchical diagnosis model based on the fault tree analysis was proposed (in addition to the logical and sequential diagnosis model) to improve the fault diagnosis procedure. A completely separate type of approach that models the control process by using a distinct class of automaton, called Nondeterministic Autonomous Automaton with Output (NDAAO) was introduced by Klein et al. [8]. There are many research articles which later extended that basic work (recent examples include: [9–13]). In these types of approaches, the nominal manufacturing process behaviour is represented by a NDAAO type automaton and the faults are detected based on whether the observed system behaviour is following the transition behaviour of the NDAAO type automaton. In [14, 15], a (deterministic) Finite State Machine (FSM) based approach was proposed for fault detection. In another paper [16], the P-invariant of Petri Nets was applied to discover the sequence faults and the exclusive logic functions were applied to detect the sensor and actuator faults.

Unfortunately, all the above-mentioned approaches are focused only on the theoretical justification of fault detection procedure, and behaviour anomaly detection has not been considered. In addition, they actually used the log data records from a small manufacturing process (or a small PLC control program) in order to validate their approach. These days, in a manufacturing system, a PLC is responsible for controlling thousands of devices and each device is operated by potentially many signals. Millions of signals performing billions of input-output operations take place every day. To find faults, it does not suffice to have only a theoretical or mathematical framework. It is also necessary to have an efficient software implementation of that framework; thus the real time identification of faults (or behavioural anomalies) becomes possible with feasible computer resources. All the mentioned literatures mainly focus on theoretical framework design and practical implementation details or empirical results of their timeliness have been less stressed. Moreover, all the above-mentioned approaches use a single state of their theoretical control process model to represent the complete physical state of the manufacturing system. These types of approaches become infeasible with growing complexity of the manufacturing system. The approaches proposed in [6, 16] do not fall into this category since they utilize the PLC code for fault detection. However, approaches like this require additional PLC scanning time and, therefore, increase the difficulty to achieve fast responses in real time control (especially for large manufacturing systems).

In this paper, we present an automated behavioural anomaly and fault detection tool called PLAT that can overcome the above-mentioned limitations. PLAT automatically creates a model of the manufacturing system control process and finds faults and behavioural anomalies using that model. PLAT uses the log data records of the PLC signals for this purpose. Actually, rapid advances in communication technology have dramatically improved the timeliness and accuracy of the PLC signal log data records. These days, data loggers are able to produce highly accurate log data records of the PLC signals in real time (see, e.g., [17–19]). Thereby log data records of PLC signals became a useful source of information and opportunities have been created for researchers to detect faults and behavioural anomalies in real time by analyzing these log data records. Our proposed tool PLAT can easily identify faults and behavioural anomalies from the log data produced by a manufacturing system in real time. Moreover, without requiring process specific control knowledge, PLAT can provide an easy understandable graphical representation of the complicated device control behaviour to operators. PLAT can also handle a large manufacturing system with reasonable computer configuration. The remainder of this paper is organized as follows: the control process modelling and the working procedure of PLAT are described in Section 2. Results of our experiments are presented in Section 3.  Section 4 contains our conclusive remarks of the work followed by a list of relevant and state-of-the-art references.

## 2. PLAT: Control Process Model Design and Working Procedure

This section is divided into six subsections. In Section 2.1, the problem description is given. An overview of the PLAT system and the theoretical control process modelling procedure are discussed in Sections 2.2 and 2.3, respectively. In Section 2.4, the control process model implementation procedure of PLAT is described. The control process model indexing mechanism (in computer memory) and the fault and behavioural anomaly detection procedure of PLAT are presented in Sections 2.5 and 2.6, respectively.

### 2.1. Problem Statement

**Figure 1:**
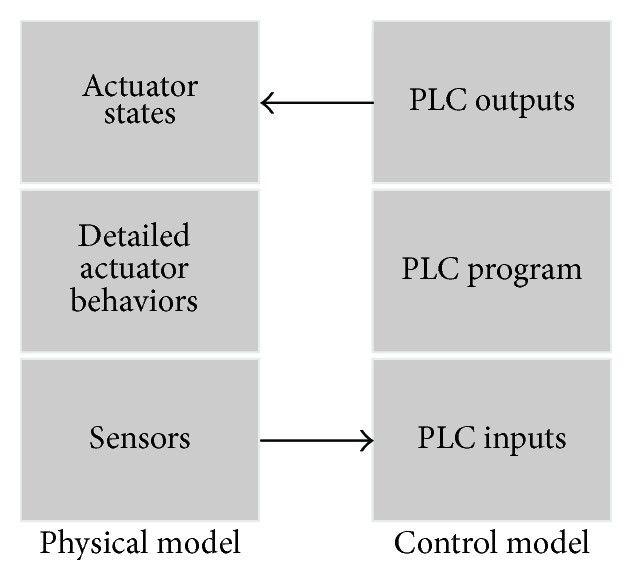
PLC controlled manufacturing system model.

In Figure 1, an overall model of the PLC controlled manufacturing system is given. PLC controls the manufacturing system according to the control program embedded in its controller. As can be seen in Figure 1, after execution of the PLC program, it supplies the output information (by converting them into electrical signals) to the actuators such as motor starters and switches. Actuator operations take place and the resulting new sensor signals that identify the state of the control process are transmitted back to the PLC program. In the PLC memory, these discrete state PLC input-output (I/O) signals indicate the operating states of the manufacturing system by which behavioural anomaly and fault detection can be carried out. The data logger continuously records these discrete I/O signal data sets from the PLC memory and inserts them into the log database for further analysis.

**Figure 2:**
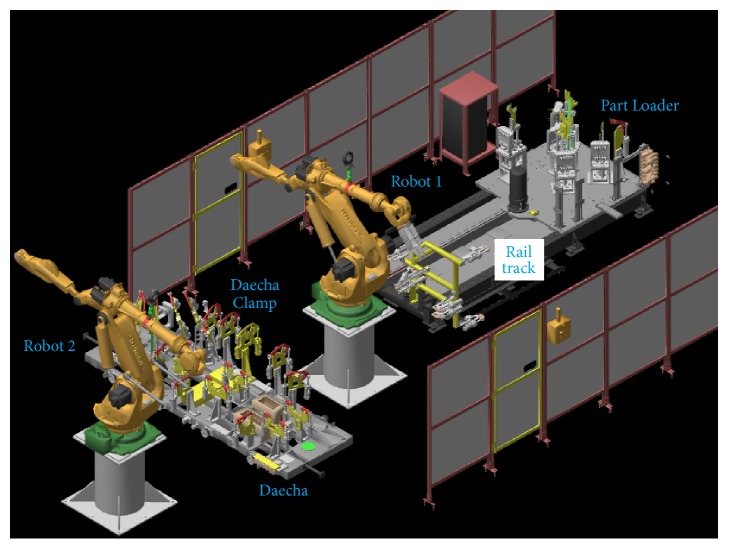
Manufacturing subsystem: group of devices.

Every PLC signal log data record contains three fields of information: (i) time stamp, system clock time when the log data record was generated; (ii) symbol name, formal PLC signal name; and (iii) value, which represents the ON/OFF status of the signal. An example log data format is given in Table 1. The time stamp is given in hour, minute, and second format (separated by a “:”). The symbol name provides the PLC signal name extended by the device (that the signal operates) and the group name to uniquely identify that signal (in Table 1, signal, device, and group names are separated by a “-”). PLC controlled manufacturing systems are modular systems; that is, they are composed of many subsystems that operate together to fulfill a composite task. Usually, devices that work together for a particular job form a group in the manufacturing system (i.e., independent subsystems of the manufacturing system). An example group of a manufacturing system is shown in Figure 2. As can be seen, this subsystem is completely independent and isolated. In order to apply our PLAT tool, symbol names must follow this specific naming rule (as shown in Table 1). The naming convention of symbol name may differ according to system users' preferences. However, it is not difficult to change it to the required format stated above (can also be implemented by providing an additional PLC address to symbol name mapping module). The field name “Status” of Table 1 provides the ON/OFF status of its corresponding signal (1 for ON and 0 for OFF). PLC data logger repeatedly examines the PLC memory and inserts the log data record into the database whenever a signal changes its status value (see, e.g., [17]). A fault occurs in the control process, if any signal is found in an erroneous state (i.e., instead of an ON state, an OFF state is observed, and vice versa) and behavioural anomalies take place, if significant change in any signal (or device) behaviour is observed. In the manufacturing industry, daily billions of log data records are inserted by the data logger into the rapidly growing database for future analysis and, hence, an efficient data handling mechanism is needed in order to identify faults and behavioural anomalies in real time.

### 2.2. PLAT System Overview

**Figure 3:**
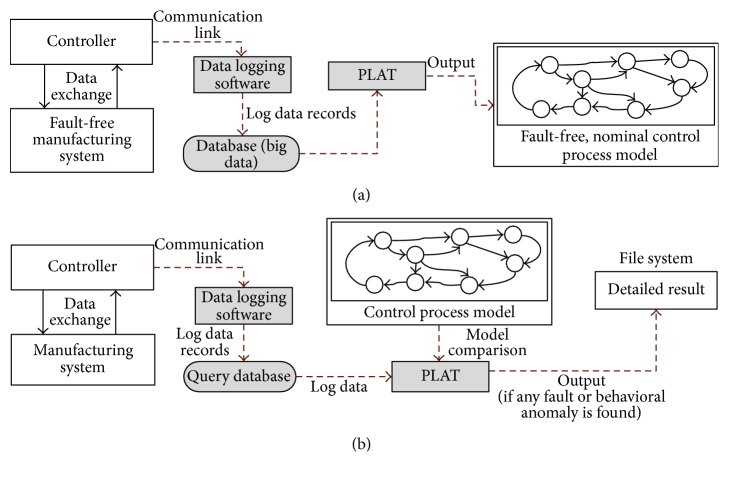
PLAT system overview. (a) Control process model generation. (b) Fault and behavioural anomaly identification procedure.

An overall model of the working procedure of PLAT is given in Figure 3. As can be seen from Figure 3(a), at first a nominal model of the PLC control process is built by using the log data records taken from a fault-free manufacturing system. In literatures, it is often argued that it is hard to find a fault-free manufacturing process in real life. Actually, we have used the term “fault-free” to denote the acceptable one. This makes it possible that some soft faults are assimilated in the control process model [in PLC terminology, the term “soft faults” refers to the faults that are unknown to the system and are not fatal to the system operation]. However, since the model contains the soft faults in an acceptable range, it is always possible to identify the real fatal one. Moreover, the presence of low number of soft faults also helps to avoid a huge number of unimportant soft fault detections (in this paper, the term “fault” actually refers to the hard or fatal fault; the same is also true for the behavioural anomalies). The fault and behavioural anomaly identification procedure of PLAT is shown in Figure 3(b). As can be seen, the nominal control process model is compared with the observed log data records in order to detect the faults and behavioural anomalies. If there is no difference between the observed and the expected behaviour of the signals, then the system is supposed to be fault or behavioural anomaly-free. Otherwise, the detailed information about the fault or the behavioural anomaly is reported to the file system. We have used Process Optra [20] data logger (customized for our purpose) to acquire the log data records from PLC memory.

### 2.3. Control Process Model Design

This section is divided into two subsections. The control process modelling mechanism of PLAT is given in Section 2.3.1 and the theoretical foundations of the model are discussed in Section 2.3.2.

#### 2.3.1. Theoretical Control Process Model Formulation

**Figure 4:**
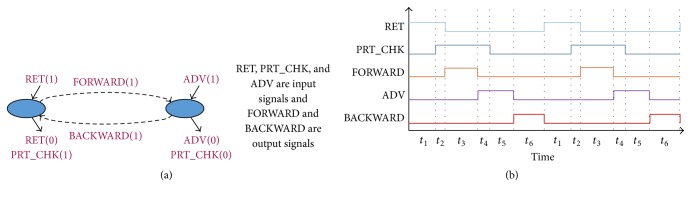
Part Loader device behaviour. (a) State based I/O model. (b) Signal-time chart.

In a PLC controlled manufacturing system, a set of PLC I/O signals are used to control a particular device. In literature, PLC controlled device behaviour is usually represented by a state-based I/O model or by using a similar kind of automaton model [6, 21, 22]. A state-based I/O model of a device actually expresses the functional role of its control signals. We discuss the device behaviour modelling by using the Part Loader device example of Figure 4 (see Figure 2 also). In the initial phase, a part is loaded to the Part Loader. Then, it moves through rail track towards Robot 1. After reaching the end position, Robot 1 picks up the part from it and, then, the Part Loader returns back to its home position. In Figure 4(a), state-based I/O model of that Part Loader device is given (see, e.g., [21]). As can be seen, Part Loader is operated based on five control signals. Among them, the RET and the ADV input sensor signals are used to notify the location of the device (i.e., Part Loader is in home or advanced position), and the PRT_CHK input sensor signal is used to notify if a part is loaded in the Part Loader. Two output signals, that is, FORWARD and BACKWARD, are used to operate the Part Loader field device. An example signal-time chart depicting the behaviour of its control signals is given in Figure 4(b). In the initial state, the process starts when the Part Loader is in its home position. So, the sensor at the home position detects it and the RET signal becomes ON. If a part is loaded to the Part Loader, then the PRT_CHK signal is turned ON. This causes the Part Loader to move towards the Robot 1 by making the FORWARD signal ON, and after this motion is started, the RET signal becomes switched OFF. If the ADV signal is turned ON that means the Part Loader has reached the end position of the rail track, and this causes the FORWARD signal to be turned OFF. The PRT_CHK signal goes to OFF state when Robot 1 picks up the part and then Part Loader starts to return to its home position by making the BACKWARD signal ON. This causes the ADV signal to be turned OFF. The RET signal becomes ON when the Part Loader returns to its home position and after this the BACKWARD signal becomes switched OFF. This cycle, called device cycle, starts again when another part is loaded to the Part Loader. The state-based I/O model of Figure 4(a) depicts this behaviour. The small arrows around the circle signify when the model enters or leaves that specific state. The transition FORWARD(1) specifies that the FORWARD signal is ON and represents the forward motion (towards Robot 1) of the Part Loader. Similarly, the BACKWARD(1) transition represents the backward motion (towards the home position) of the Part Loader.

**Figure 5:**
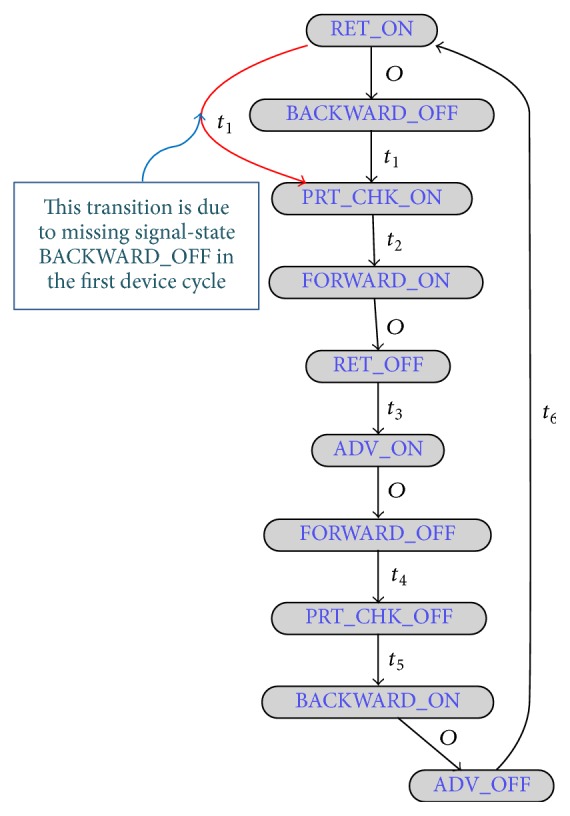
Signal-state I/O model of the Part Loader device.

We can easily define this entire device behaviour (as can be seen in Figure 4) using signal-state I/O model of Figure 5. With this signal-state I/O model, we are able to incorporate all the behavioural information of the Part Loader stated above. Actually, the state-based I/O model of Figure 4(a) is simply redrawn to consider the signal-state transition sequence information. For example, in Figure 5, Part Loader starts working when the RET signal is ON, represented by the RET_ON signal-state (signal name extended by its state). At the same time, the BACKWARD signal is needed to be switched OFF and, hence, the next signal-state of the Part Loader is the BACKWARD_OFF. Then it moves to the next signal-state which is PRT_CHK_ON, and so on (see Figure 4(b) also). The transition from RET_ON to PRT_CHK_ON signal-state (shown in red color) occurs due to the missing BACKWARD_OFF signal-state in the first device cycle (as no signal status change event takes place in the BACKWARD signal). We can easily identify it from the number of transition occurrences. We have also incorporated the transition time information in this model (signal-time chart information of Figure 4(b)). The values associated with the transition arrows in Figure 5 specify the time that corresponding transitions should take. This compact theoretical representation based on event-sequence information of the I/O signals corresponding to a device not only helps PLAT to detect faults and behavioural anomalies but also helps it to gain speed during fault searches.

**Figure 6:**
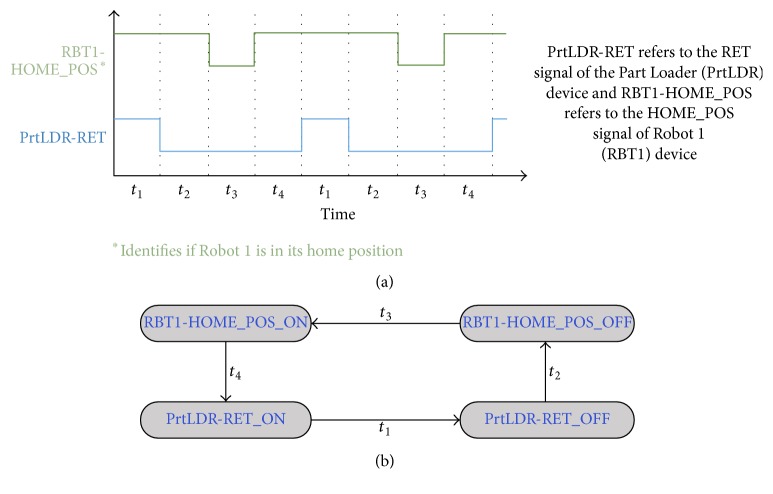
Modelling group behaviour. (a) Signal-time chart of the starting signals of the devices. (b) Signal-state I/O model of the group.

Our main motivation is to model all PLC controlled device behaviour using this kind of signal-state I/O model as shown in Figure 5. The signal-state I/O model expressed in terms of a device's control signals actually defines its control characteristics. If any device behaviour fails to follow its corresponding signal-state I/O model, then we can easily conclude that faults or behavioural anomalies have taken place in the control process. Faults in the signal-states (or changes in the signal behaviour) will also affect the signal-state I/O models of the devices, by which we can easily identify faults (or behavioural anomalies). For example, after ADV signal goes to OFF state (notifies Part Loader is returning back to its home position), if we find that the PRT_CHK signal becomes ON again due to some unexpected PRT_CHK signal behaviour, then we can easily conclude that the fault has taken place in the control process. In that case, we will get a transition error while moving from ADV_OFF to PRT_CHK_ON signal-state (faulty transition, according to the model in Figure 5). We must build such signal-state I/O model not only for each device but also for each group; thus interrelations between device control processes can be defined. For example, as we have stated earlier, the part is removed from the Part Loader by Robot 1 after reaching the end of the rail track (see Figure 2). This information needs to be included in the control process model and, hence, signal-state I/O models for the groups are also required (recall that devices that work together form a group). Modelling of group behaviour is similar. A device is represented by its starting (or any other) signal in the signal-state I/O model of its group. The starting signal of a device refers to the first signal that appears in its signal-state I/O model (in our Part Loader example, the RET signal is the starting signal). For simplicity, if we can think of a device as having only one signal, that is, its starting signal, and a group as a device, then the modelling of the group can be the same as the device. As an example, Figure 6(a) shows signal-time chart of the starting signals of two devices (i.e., Part Loader and Robot 1) of the group presented in Figure 2 (the signal name is extended by the device name to uniquely identify that signal in that group). The signal-sate I/O model representation of that group is presented in Figure 6(b) (assume that the group of Figure 2 contains only those two devices). As we can see, at the beginning, both starting signals of that group are ON at the same time. However, this happens only for the first device cycle. From the second device cycle onwards, RBT1-HOME_POS_ON to PrtLDR-RET_ON transition takes time *t*_4_ (which is actually the true transition time) and, hence, only the transition time *t*_4_ is shown. The signal-sate I/O models of the groups are also required for fault detection. This is because some of the devices are operated based only on very few input signals and, hence, if any or some of those signals go to ON/OFF state permanently due to the fault, the device may stop working (which means no signal status changes can be observed for that device). In that case, PLAT can identify the fault from the signal-sate I/O model of its group. We do not build any model to define the interrelation between operations of groups because, as we have stated previously, groups are independent subsystems that have no relation to each other. Our complete control process model is a composition of each signal-state I/O model of the devices and groups (with some small modifications, described in next paragraph).

**Figure 7:**
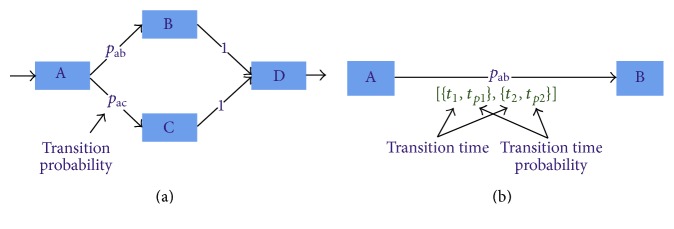
Modified signal-state I/O model. (a) Signal-state I/O model with multiple transitions. (b) Modified signal-state transition model.

The signal-state I/O model of a device or group will not always be as simple as depicted in Figures 5 and 6(b). In the signal-state I/O model, one state may have multiple transitions (as shown in Figure 7(a)) and each transition may have multiple transition times (as shown in Figure 7(b)). The multiple transitions or multiple transition times not only can occur due to some missing status change events of the signals in the first device cycle but also can take place depending on the manufacturing process condition at that particular time point. For example, consider a scenario where a robot picks a part from the Part Loader and place it into two bins according to its quality (say, good or bad). The signal-state I/O model of that robot can have two different transition paths according to the quality of the part. Now, suppose that if the quality of the part is good, then it will be processed further and if it is bad, then the Part Loader returns back to its home position immediately to carry another part. In this scenario, the PRT_CHK_OFF to the BACKWARD_ON signal-state transition (see Figure 5) has two transition times, one for good and another for bad quality parts. In the signal-state I/O model, if we deal with multiple transitions (or multiple transition times) by simply adding another path to the corresponding signal-state node, then we miss some information such that (suppose) the percentage of bad quality parts is much lower than the percentage of good quality parts (if it becomes high then that could be an anomaly in the control process behaviour). We incorporate this kind of information in signal-state I/O model by including the state transition probabilities as shown in Figure 7(a) (the edge values shown are transition probabilities; for clarity, transition times are omitted). A transition with multiple transition times can be thought as different transitions and each such transition certainly has a transition probability that is simply incorporated into Figure 7(b). So, in our modified signal-state I/O model, each transition has two kinds of information attached to it: (i) transition probability and (ii) transition times and their corresponding probability.

#### 2.3.2. Theoretical Foundations of the Signal-State I/O Model

A signal-state I/O model of a device or group is actually a kind of discrete-time Markov chain [23, 24] (see the following definitions).

**Definition 1 .**

Suppose, {*X*_*n*_,   *n* ≥ 0} is a discrete-time stochastic process that can take values from a countable set *S*, called the state space. {*X*_*n*_, *n* ≥ 0} is called a discrete-time Markov chain (DTMC) if it satisfies the Markov property. The Markov property can be formally expressed in the following way:

(1) PXn=in ∣ Xn−1=in−1,Xn−2=in−2,…,X0=i0=PXn=in ∣ Xn−1=in−1∀n≥0,  in,in−1,…,i0∈S,

where *P*(*X*_*n*_ = *i*_*n*_) is the probability to be in state *i*_*n*_ at step *n*

In other words, the conditional distribution of the future states of DTMC depends only on the current state and is independent of all the previous states (see, e.g., [23, 24]).

**Definition 2 .**

A DTMC is said to be time homogeneous, if *P*(*X*_*n*_ = *j*∣*X*_*n*−1_ = *i*) = *P*_*ij*_ is independent of *n*.

**Definition 3 .**

The probability of the transitions from state *i* can formally be defined as follows: ∀*ij* *P*_*ij*_ ≥ 0 and ∑_*j*∈*S*_ *P*_*ij*_ ≡ 1  [∀*i*, *j* ∈ *S*].

So, if we exclude the timing information from the model, then the signal-state I/O model (of a device or a group) is basically a time homogeneous DTMC (its state space is composed of the signal-status names of all the signals belonging to that device or group). Please note that the time homogeneity and the Markov property are satisfied because the system states are changed according to the sensor feedback to the PLC program (dynamic execution; see Figure 1).

**Definition 4 .**

Let *P*_*ij*_^*n*^ = *P*(*X*_*k*+*n*_ = *j*∣*X*_*k*_ = *i*); then state *j* is said to be reachable from state *i*, if there exists *n* ≥ 1 such that *P*_*ij*_^*n*^ > 0. The state *j* is said to be one-step reachable from state *i*, if *P*_*ij*_^1^ (or, simply, *P*_*ij*_) >0.

If a fault occurs in the control process, it completely alters the sequence of status change events of the PLC signals (in other words, the signal-state transition sequences of the devices and groups are completely modified) [10–14]. So, if PLAT finds any signal-state of a device or group that is not one-step reachable from its immediate previous signal-state (according to the corresponding nominal signal-state I/O model), then we can easily conclude that a fault has taken place in the system. In some extreme situations, the whole manufacturing subsystem (or group) may stop working because of the occurrence of a fault (implying that the corresponding control program has fallen into an unproductive loop). For example, if a failure in PRT_CHK sensor occurs at the beginning of a Part Loader device cycle (see Figures 4 and 5), then the subsystem of Figure 2 stops working (because Part Loader cannot transfer the part to Robot 1). These types of faults can easily be identified by maintaining a timer counter in computer memory for each group [if any timer gets expired (after a relatively long time period) that implies a fault has occurred in the control process]. PLAT automatically builds the signal-state I/O models (with modified transition model, as in Figure 7(b)) for the devices and groups and attempts to find faults and behavioural anomalies using those models. PLAT can identify two types of behavioural anomalies: (i) transition time error (if a transition takes longer than anticipated) and (ii) transition or transition time probability error (if any significant changes in probability occur).

To summarize the above discussion, we model the control process in terms of the behaviour of the groups that it operates. To define each group behaviour, first we characterize each device behaviour of that group in terms of the interrelations among its control signals and, then, we characterize that group behaviour in terms of the interrelations among the starting signals of those devices (a kind of divide and conquer strategy is taken). We took this design approach because defining group behaviour in terms of the interrelations among all the control signals in that group (most of the existing approaches use this strategy) could generate a very complex model with many high-degree signal-state nodes. For example, suppose a group contains two Part Loader devices and their independent actuators act exactly in parallel. If we define group behaviour in terms of the interrelations among control signals, a small delay in one of the Part Loader signal-state transitions could generate a very complex signal-state I/O model with many unnecessary transition edges. A goal of our approach is to reduce the total number of transitions (or total degree of the graph) required for modelling the group behaviour by using device-level abstraction. This gives us speed gain during fault and behavioural anomaly searching phase (by significantly cutting down the size of the model). In PLAT, a fault (or an anomaly that has significant impact on system operation) cannot remain unidentified because, in that case, the system fails to produce the exact same sequence of transitions (with almost the same transition time, transition, and transition time probabilities) as in the nominal signal-state I/O models [11, 12, 14] [we should mention that most of the existing automaton (see for instance: [9–14]) and event-sequence based approaches (see, e.g., [2, 3]) also provide the same fault detection accuracy rate, as they use the same fault detection principle (i.e., detect the faults based on whether there is any deviation in the sequence of signal status change events); anyhow, they do not solve the complete problem of real time fault and behavioural anomaly detection in large manufacturing systems]. So, the above stated model-size reduction is done without losing any useful information related to faults or behavioural anomalies associated with control process. Moreover, device-level abstractions give system users easily understandable domain and control process error interpretation. Unlike PLAT, the existing approaches cannot deal with large manufacturing processes (see Section 1). As an example, consider the NDAAO automaton based approaches [8–13]. A single state of the NDAAO automaton characterizes the complete manufacturing process state and is represented by a boolean vector of length *N*, where *N* is the total number of PLC I/O signals (means, theoretically it is possible to have 2^*N*^ number of states). This boolean vector is needed to be packed in a very few computer words through hashing (otherwise, it will take long computational time during fault searches). If the vector length *N* is very high then it can generate a large number of hash code collisions that makes it practically infeasible [note that a faulty state can take any value from 0 to (2^*N*^ − 1)]. Unfortunately, there is no straightforward way to handle this (the same argument is also valid for the other automaton and event-sequence based approaches mentioned in Section 1). The maximum number of possible states in our proposed control process model is comparatively quite lower, that is, 2 × (*N* + *M*), where *N* is the total number of signals and *M* is the total number of devices. In addition, as all the state names can be obtained in advance (using PLC program symbol list), the hash code collisions can easily be avoided with a reasonable collision resistant hash function and an appropriate seed value (in case of other automaton based approaches, it is impossible to obtain the identification number or name of the faulty state in advance). At this point, we should mention that some large manufacturing systems are controlled by multiple PLCs and are informationally centralized. In a centralized architecture, there exists a main server computer where log data records from multiple sources are gathered. Unlike other approaches, PLAT can easily handle such informationally centralized systems. This becomes possible because the complete control process model of PLAT is built based on the signal-state I/O models of the devices and groups (not based on the complete process states).

### 2.4. Control Process Model Implementation Procedure

This section has two subsections. In Section 2.4.1, the signal-state transition model (a signal-state I/O model without transition time, transition, and transition time probabilities) building procedure and the transition probability calculation procedure of PLAT are presented. In Section 2.4.2, we discuss the procedure used by PLAT to find the transition times and their corresponding probabilities.

#### 2.4.1. Signal-State Transition Model Building and Transition Probability Calculation Procedure

**Figure 8:**
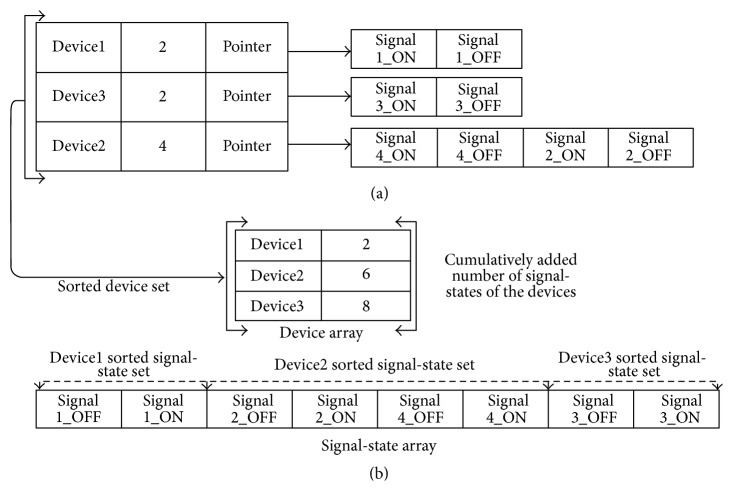
Data structure for storing signal-state information of a group. (a) Data structure for storing signal-state information of the devices of a group. (b) Converted sequential data structures.

PLAT automatically builds the signal-state I/O model for each group and device from the PLC signal log database. The log database must be from a fault-free system (see Figure 3(a)). PLAT starts working by collecting all the device names and their corresponding signal-state names (using PLC program symbol list) for each group by using the data structure of Figure 8(a). In this data structure, first column stores the name of the devices, second column stores the number of signal-states associated with that device, and third column stores the corresponding set of signal-state names through a pointer to an array (an arrow represents a pointer access). For each symbol name, the device name of that record is inserted into the first column of its corresponding group's data structure, and the signal name, extended by its state, is inserted into the signal-state set of the corresponding device through a pointer access (corresponding second column value will be updated accordingly). Then the data structure of Figure 8(a) is converted into two sequential data structures, as shown in Figure 8(b); thus the need to access signal-state set through the pointers can be eliminated (for speed gain). The conversion procedure is simple. The first column of the Device Array in Figure 8(b) contains the sorted set of device names stored in Figure 8(a). The second column values of the Device Array are the cumulative addition of the number of signal-states of the devices. The signal-state array of Figure 8(b) simply contains all the signal-state names of all the devices stored in Figure 8(a). The signal-state set of each device is inserted into the signal-state array according to the order of devices in Device Array (the signal-state set is sorted before insertion). With these sequential structures, mapping a device to its corresponding signal-state set (reverse mapping) is simple. For example, in the signal-state array, the signal-state set of Device2 starts from position 2. This position is recorded in the second column corresponding to the previous row of Device2 in Device Array (this position for the first device name in Device Array is 0). The number of signal-states belonging to Device2 can be calculated from the Device Array by subtracting the position number (i.e., 2) from its corresponding second dimension value (i.e., 6). This explanation shows that, with these sequential structures, we can easily access the complete signal-state set that can contain a large number of elements without any pointer access (just one additional subtraction is needed). Moreover, as the signal-state set of each device is sorted, we can easily perform binary searches on it.

**Figure 9:**
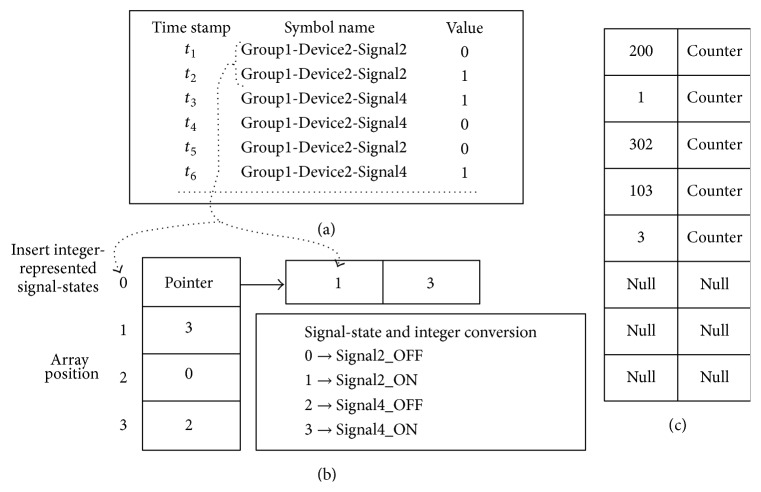
Signal-state transition model building procedure. (a) Example log data records of Device2. (b) Data structure for signal-state transition model creation. (c) Hash table structure for calculating transition probabilities.

The signal-state transition model building procedure is depicted in Figure 9 based on the example given in Figure 8. At first, the log data records are sorted according to the device name of each group (which means log data records related to the first device name of the Device Array are processed first and so on; this process is repeated for each group). An example set of log data records (suppose Device2 of Figure 8(b) and Device2 belong to Group1) and its corresponding signal-state transition model are given in Figures 9(a) and 9(b), respectively. A signal-state is represented in the data structure of Figure 9(b) by its position number in the signal-state set of the corresponding device. For example, in Figure 8(b), Signal2_OFF is the first signal-state in the signal-state set of Device2 and, hence, is represented in Figure 9(b) by 0. Recall that the signal-state set of each device is a sorted set and, hence, integer conversion can be done by performing a binary search. In Figure 9(b), the array position and corresponding array value(s) are basically integer-represented signal-states that indicate between which signal-states the transition happened. The data structure of Figure 9(b) is an array initialized with null values, and any integer values representing signal-states may simply be inserted into the corresponding array position. If the corresponding array position is already filled, then it is replaced by a pointer to an array, and the old value(s) and the new value are inserted into that new array. In general, a large number of signal-states do not have multiple transitions; thus this kind of data structure will give speed gain by significantly reducing pointer accesses. We converted signal-states to integer values; thus we can largely avoid expensive string comparison operations. While building such a signal-state transition model, we do have to maintain a hash table for calculating the transition probabilities. This hash table, shown in Figure 9(c), is a simple two-dimensional array. In the first dimension, the hash code of the signal-state transition is stored and, in the second dimension, a counter is maintained which identifies how many times that transition has occurred.

The hash codes of the signal-state transitions are generated by using a simple hash function, that is, (Current_State × *q* + Next_State), where “Current_State” and “Next_State” are integer-represented signal-states between which transitions have occurred, and *q* is an integer number greater than the number of signal-states for that device. In Figure 9(c), the *q* value is considered 100 (assuming the number of signal-states are less than that). This simple hash function distributes the hash values quite well over a large output domain (in every interval of size *q*, there will be very few instances) and there will be no chance of hash code collision (because each signal-state is represented by a unique integer number). For collision resolution in the hash table (because the hash codes are mapped onto a small number of buckets by modulus operation), we use simple linear probing technique [25]. This is because any complex collision resolution technique could penalize performance as our hash function has already uniformly distributed the key-values over the bucket range. Signal-state transition models for the groups are also created following the same procedure. However, some additional modification is required for the signal-state to integer conversion procedure in order to maintain uniqueness of the integer-represented signal-state values. The signal-states of a group are integer-represented by the following:
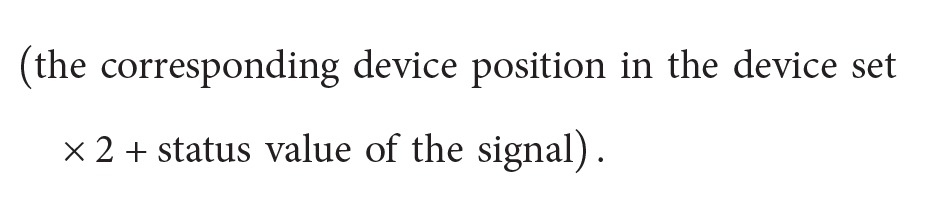
(2)


For example, in Group1, signal-state Signal2_OFF of Device2 (belonging to the starting signal of Device2) is represented by (0 × 2 + 1) = 1 [recall that the value of status OFF is 0 and the position of Device2 in Device Array of Figure 8(b) is 1]. This simple conversion provides unique signal-state representations for a group (because a device is represented by its starting signal in the signal-state I/O model of its group). By using this simple procedure, signal-state transition model for a group or a device is built and transition probabilities are calculated.

#### 2.4.2. Transition Time and Transition Time Probability Calculation Procedure

We now discuss the procedure used by PLAT to find transition times and their corresponding probabilities. Recall that, in our previous signal-time chart example of a Part Loader (see Figure 4(b)), the signals are always ON or OFF for a fixed amount of time. For example, the FORWARD signal is always ON for time *t*_3_. In practice, this is not always the case. As can be seen in Figure 4(b), the FORWARD signal is ON until the Part Loader reaches the end of the rail track. This time depends on the speed of the physical device (as sensors give feedback to the PLC program; see Figure 1) and, hence, cannot always be the same. This can also vary depending on the time stamp accuracy of the data logger. As can be seen from Figure 5, for this reason, the transition time instances for a particular transition will also vary. However, they do not vary significantly because of the almost fixed speed of the physical device and modern, highly accurate data loggers [17, 18]. We can represent this transition time by taking the mean of these instances (the average transition time). However, as stated before, a transition can have multiple transition times. In a large manufacturing system, where thousands of devices are working, it is difficult to know how many transition times a particular transition actually has and, hence, in order to determine this, we need a transition time clustering algorithm. The purpose of clustering is to separate the time instances for a particular transition into several clusters; thus classification of the time instances (as “normal” or “erroneous”) can be done by choosing a representative point from the clusters. In our case, the cluster means (average transition times) are used as the “actual” transition times in the signal-state I/O models and the highest-valued instances of the clusters (maximum transition times) are used to determine the transition time errors.

Several approaches have been introduced in the literature to address the above-mentioned clustering problem, such as Jenks natural breaks, *k*-means, PAM, CLARANS, AGNES, DIANA, CURE, and DBSCAN [26–34]. In general, the time complexity of these clustering algorithms is very high. For example, all the mentioned algorithms (except *k*-means) have a time complexity ≥*O*(*n* ^2^), where *n* is the total number of instances [26–34]. With the access of special data structure, the time complexity of these algorithms can be reduced. However, often construction of such data structure severely penalizes the performance [31]. We have to perform clustering for each transition and this can severely penalize the performance if we employ an algorithm with high computational complexity (or with a special data structure construction cost). Unlike others, *k*-means algorithm has a very low time complexity of *O*(*tkn*), where *t* is number of iterations, *k* is number of clusters, and *n* is number of instances (usually *k*, *t* ≪ *n*) [29–31]. Moreover, *k*-means algorithm is a widely used approach in the literature because of its very fast software execution. In recent research, *k*-means algorithm has been widely used for clustering one-dimensional data for these reasons as well [35]. However, *k*-means algorithm has one well-known weakness; that is, it often terminates at local optima. This issue can largely be avoided by using careful seeding technique [36]. Several seeding methods have been proposed in literature; however, they mainly focus on high dimensional data. In fact, one-dimensional data is easy to handle (particularly in our case) as it can be easily sorted (we always use Introsort algorithm [37] for this purpose) and its distances can be easily calculated. Hence, different seeding technique is required to take advantage of one-dimensional transition time data.  Algorithm 1 provides our overall algorithm for finding average and maximum transition times of the clusters and their corresponding probabilities. The algorithm returns the mean and the highest value of the transition times if its instances do not vary much (which implies single cluster; see Algorithm 1, lines (3)–(10)). Otherwise, *k*-means algorithm (with a careful seeding strategy) is applied (see lines (12)–(34)). The seeding technique of our *k*-means algorithm is inspired by Jenks natural breaks method [26–28]. Informally, Jenks natural breaks method attempts to find “breaks” (positions from where new cluster should start) in the sorted number line where most significant gaps (or differences) appear [26–28]. The definition of “significant gap” varies from lower- to higher-order numbers. We have incorporated this intuition into our seeding technique and definition of gap in our algorithm (see lines (12)–(21)). In fact, we have just calculated the relative differences of the data points in our gap calculation that indeed penalizes the higher-order numbers. We have introduced* parameter  ε* to penalize very low order numbers (this also prevents any “division by zero” errors; see line (14)). The* parameter* *ε* is not difficult to set and should be set to a small integer number (further details are in our experimental section). The mean values of the partitions (partitioned by those breaks) are supplied as initial cluster centers to the *k*-means algorithm to overcome the class outlier sensitivity issue (see lines (22)–(34)). For the convenience of the readers, the detailed *k*-means algorithm is presented in Algorithm 2 [the Introsort algorithm is not given here for lack of space; it can be found in [37], pp. 986–987].

The main goal of our seeding technique is to spread out the initial cluster centers according to the data distribution; thus the poor local optima problem can be largely avoided. Though theoretically our careful seeding technique does not guarantee optimality, in practice, it binds the local optima (if global optima are not found) very close to the global optima, because of the initial cluster center choices. In practice, we have also seen that usually the transition time clusters are well separated (can be realized from our discussion of Section 2.3) and, hence, it is relatively easy for our simple *k*-means algorithm to bind the local optima very close to the global optima. We call our proposed *k*-means algorithm carefully seeded *k*-means (CS*k*-means algorithm). CS*k*-means algorithm is *O*(*n*log⁡*n*) competitive with the traditional *k*-means algorithm. This is because we use Introsort algorithm (see Algorithm 1, line (4)) with time complexity of *O*(*n*log⁡*n*) for sorting the data points [please note that the gap calculation procedure of CS*k*-means algorithm (see Algorithm 1, lines (12)–(21)) also gives additional time complexity of *O*(*kn*); however, it is compensated during the *k*-means algorithm (see Algorithm 2, lines (1)–(25)) because the *k*-means algorithm is performed on the sorted transition time data]. After CS*k*-means algorithm, the (single-linkage) Hierarchical Agglomerative Clustering (HAC) algorithm [29, 38] is applied to merge the clusters whose centers are not “far away” (in a user-defined way) from each other (see Algorithm 1, line (35)). For reader's convenience, the HAC algorithm is provided in Algorithm 3. Usually, transitions have very small number of transition times and we recommend using slightly higher value as the *k* value (*k* is the number of clusters) because this will help CS*k*-means algorithm to dynamically mitigate the outlier issue (i.e., for an outlier CS*k*-means algorithm will not lose an actual cluster). In the manufacturing systems, the transition time outliers mostly originate during the system start-up time. This is the reason why we discard clusters that have very low support (see Algorithm 3, lines (36)–(41)).

The signal-state I/O models of the devices and the groups are build using this procedure (as discussed in Section 2.4.1 and in this subsection) and are saved into the file for future use. The integer-represented signal-states are converted back to their original form (i.e., signal-state name extended by its device and group name format). For user's convenience, signal-state I/O models are saved in DOT language (a graph description language) and can be visualized by any DOT language parser such as Graphviz [39]. This gives an easy understandable graphical representation of complicated device or group control behaviour to users. Moreover, maintenance engineers can easily identify detailed effects of the faults from this graphical visualization. The procedure discussed above actually helps PLAT to generate the signal-state I/O models rapidly from a large database.

### 2.5. Signal-State I/O Model Indexing Mechanism

**Figure 10:**
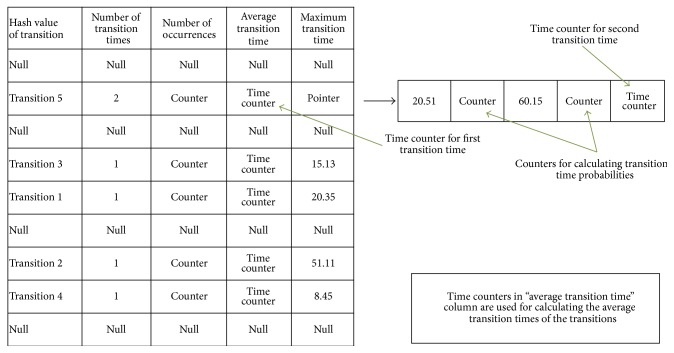
Index hash table: hash table representation of signal-state I/O models.

After building the control process model, PLAT has a set of signal-state I/O models, one for each device and group. As we have stated earlier, even for a small manufacturing system, the set can contain hundreds of such signal-state I/O models. The main challenge is to efficiently represent those signal-state I/O models in computer memory such that the identification of faults and behavioural anomalies can be done in real time. From the computer science point of view, this problem can be thought as a mapping of two databases: one database, that is, reference database from which the signal-state I/O models are built, and another database, that is, query database in which we have to search the faults and behavioural anomalies. PLAT builds an index hash table from the signal-state I/O models (saved in the file system) to satisfy this purpose. The signal-state I/O models are nothing but a series of transition rules.  Table 2 gives an example set of transition rules for Device2 of Figure 9. From now on with the term “device name” (resp., “signal name”), we will refer its complete name to uniquely identify it, that is, device (resp., signal) name extended by its group (resp., device and group) name. At first, PLAT builds a hash table, the “index hash table” (as in Figure 10) from the transition rules of all the devices and groups. In the first column of the index hash table, hash values of the transitions are stored. The hash value of a transition is the concatenation of the hash values of the signal-state names between which the transition has occurred. We use MurmurHash2 hash function [40] for hashing the signal-state names because of its high avalanche effect, collision resistance, and speed [41]. The default size of the hash value of a signal-state name is (unsigned) 32 bits (which means the default size of the hash value of a transition is 64 bits). Actually, PLAT decides the size of the hash values and the seed value of the hash function in such a way; thus no hash code collision occurs (at the time of preprocessing the PLC program symbol list). This ensures that no faults can be missed due to the hash code collision. Second column of the index hash table stores the number of transition times. In the third and fourth column, a set of counters are maintained for calculating the transition probabilities and the average transition times, respectively. In the fifth column, maximum transition times of the transitions are stored; thus transition time errors can be identified. If a transition has more than one maximum transition time, then they are stored through a pointer to an array, and counters are maintained in that array so that transition time probabilities and average transition times can be calculated (time counter for calculating the average transition time is not needed for the first transition time; see Figure 10 also). In Figure 10, some example insertions of the transition rules from Table 2 are shown (for clarity, the row numbers of Table 2 instead of actual transition names are shown). The structure of index hash table's fifth column is similar to the data structure of Figure 9(b) and used for the same reason. Here we could eliminate pointers in the index hash table by converting the fifth column into the sequential structure as done in Figure 8. However, we have not done this because a very small number of transitions will have multiple transition times and, for these, the number of times will not be high. Hence, pointer access would not be reduced much. If we wish to convert the fifth column of the index hash table into a sequential structure, then we have to build it separately from the other four columns (as done in Figure 8(b)). This will cause significant increment in the number of cache misses for poor locality of reference that pointer access elimination cannot compensate (this effect is shown in our experimental section and this structure is referred to in that section as “sequential index hash table”).

**Figure 11:**
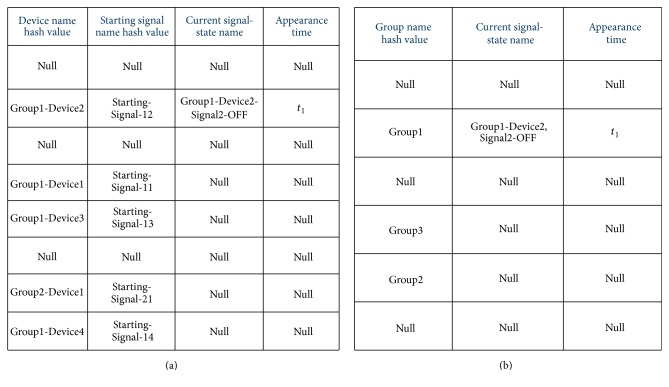
Device and group hash table structures. (a) Device hash table: hash table structure for storing signal-state information of devices. (b) Group hash table: hash table structure for storing signal-state information of groups.

After creating the index hash table, PLAT also creates another two hash tables, the “device hash table” and the “group hash table” (shown in Figures 11(a) and 11(b), resp.); thus rapid searching for faults and behavioural anomalies in the index hash table becomes possible during query database processing. Actually, in the index hash table, we store all possible transitions of all the groups and the devices. Now, in order to find a transition for a group or a device, we must store its current signal-state until its next signal-state appears in the query database. For that reason, PLAT creates these two hash tables. In the first and second columns of the device hash table, hash values of the device names and the starting signal names are stored (recall from Section 2.3.1 that a device is represented by its starting signal in the signal-state I/O model of its group). Similarly, the first column of the group hash table stores the hash values of all the group names (the MurmurHash2 hash function [40] is used for all the hashing and the (default) size of the hash values is 32 bits). The last two columns of device hash table (third and fourth column) and group hash table (second and third column) are filled with null values. These columns will be used during the query database processing phase. After completion, these three hash tables are saved into the file system for future use. We now discuss the hash table collision resistance mechanism taken by PLAT. In the hash tables of Figures 10 and 11, we map 64-bit or 32-bit (unique) hash codes into small number of hash buckets to get the corresponding position; and this may produce many hash table collisions. Several collision resolution strategies have been introduced in the literatures such as chaining, linear probing, and double hashing [25]. In our hash table structures, each key has several corresponding values and continually accessing those key-values through a pointer would be highly expensive in case of chaining approach. Hence, we use double hashing as our collision resolution technique. In double hashing, it is expected that it will produce much less probe sequences than linear probing, which will indeed make it much faster than linear probing [25, 42, 43]. In literature it is often argued that, in practice, linear probing is a better choice than double hashing due to more effective use of cache memory [42]. Actually, in the literature, most of the time linear probing has an advantage over double hashing because (i) uniform data distribution is used which gives it fewer probing sequences (generally not true in our case) and (ii) the hash table records are very small (therefore, a single cache line contains many records) and, hence, linear probing takes advantage of more cache hits [42]. As can be seen from our hash table structures, the record length is quite large and, therefore, very few records can be fit into a single cache line. Hence, linear probing cannot take advantage of more cache hits that could compensate the performance penalty caused by a high number of probe sequences. We use double hashing function of C# 2.0 [44] for this purpose because it can be computed very fast and we have seen in practice that it gives very few probe sequences. This hash table based effective indexing of the signal-state I/O models becomes possible only because of our device-group based control process modelling approach (note that a hash table lookup or a hash table insertion operation has on average constant time complexity [25]).

### 2.6. Fault and Behavioural Anomaly Detection Procedure

In this section, we discuss the procedure used by PLAT to identify the faults and behavioural anomalies in detail. Recall from Section 2.3 that if from the query database we find any transition that is not present in the signal-state I/O models (or in the index hash table), then we can conclude that fault has taken place in the control process. Similarly, if any subsystem or group stops producing the log data records for a very long period of time, then also we can conclude that a fault has occurred in the control process (identified by using the timer counters, one for each group). Please note that a fault cannot remain unidentified in this process because a system cannot generate the exact same transition sequences of the devices and groups (as in the index hash table) after the occurrence of a fault (in other words, a fault will eventually be detected since the observed faulty behaviour will eventually be distinguishable from the fault-free behaviour) [11, 12, 14]. If the transition time for a particular query transition lies below a certain threshold (user-defined, usually very small) of its corresponding maximum transition time, then we conclude that it is a “correct” transition. Otherwise, it signifies that a transition time error has occurred (the user-defined time threshold value is set in such a way; thus it assigns a larger threshold value to the transitions that have high-valued transition times). If there exists more than one maximum transition time (which implies multiple transition times; see Figure 10), then the maximum transition time instance that is most similar to the query transition time is taken as the corresponding maximum transition time (by which classification is done). The transition time property of the transitions is verified for every transition found in the query database. However, the other three properties, that is, transition probability, average transition time, and transition time probability are verified only when the system enters the steady state. For simplicity, these properties are verified periodically, after processing a large number of log data records from the query database. We should also mention that these three properties of the transitions are required to be verified according to the order of their corresponding transition positions in the complete transition sequence. This is because the preceding transitions always have an impact (especially in case of transition time behaviour) on the succeeding transitions. Some behavioural anomalies (that do not have significant impact on system operation) can remain undetected in this process (see Section 2.3 for details). This is because it is hard to set all the user-defined parameter values precisely in all the cases. However, if we set the parameter values reasonably, then the number of such undetectable anomalies will not be so high in practice.

We now discuss the actual query database processing mechanism of PLAT. In the query database processing phase, PLAT first loads all the three hash tables (the index, device, and group hash table) into memory and then it starts to process each log data record of the query database. The symbol name of each log data record is converted to the signal-state name (as done before) and then is stored (along with the time stamp) into the device hash table or group hash table until the next signal-state of that device or group, respectively, is found. The position of the signal-state name in the device hash table (resp., group hash table) can easily be found by hashing the device name (resp., group name) of that signal-state and comparing it with the first column values of the device hash table (resp., group hash table). Whether a signal-state belongs to the starting signal of the device or not can easily be identified using the second column of the device hash table. In Figure 11, an example insertion of the first log data record from Figure 9(a) is shown (suppose the log data records of Figure 9(a) form the query database). After receiving the next signal-state, the transition is searched into the first column of the index hash table. If a device or group transition is not found in the index hash table, that signifies fault has occurred in the control process. Hence, PLAT reports this in the file system, indicating between which signal-states the transition error (or fault) has occurred. The device hash table and group hash table are saved into the file system for the first identified transition error to identify the complete control process state after which a fault has occurred (this can be used by engineers along with previously saved signal-state I/O models for fault diagnosis). If the transition name is found in the index hash table, then the transition time of that query transition is compared with its corresponding maximum transition time (stored in fifth column) to identify transition time errors. We can calculate the transition time from the time stamp of the previous signal-state stored in the fourth column of the device hash table (resp., third column of the group hash table). If a transition or transition time error has not occurred then corresponding counters for calculating the average transition time, transition, and transition time probability in the index hash table are updated. After finishing this search, the columns of the device hash table (resp., group hash table) that store the previous signal-state information (signal-state name and time stamp) of that device (resp., group) are updated by the current signal-state information. This fault and transition time error searching procedure is repeated for all log data records of the query database. After processing a fixed number of log data records, PLAT periodically checks if there are any significant changes that have occurred in the average transition time, transition, and transition time probability. This checking can be easily done using the counters maintained in the index hash table and the set of transition rules (signal-state I/O models) previously reported by PLAT. It also examines periodically whether a timer counter of any group (maintained separately in an array, not shown for the sake of space) has got expired or not (the counter-reset operation is performed whenever a signal-state of its corresponding group is observed). If so, then the last observed signal-state is reported to the file; thus the engineers can easily identify after which signal-state the manufacturing process has stopped its execution. All the faults and behavioural anomalies found by PLAT are reported to the file (referring the names of the associated signal-states) for further analysis. It is easy to perceive from this discussion that the error searching procedure of PLAT has on average constant time complexity (because of the hash table searches).

**Figure 12:**
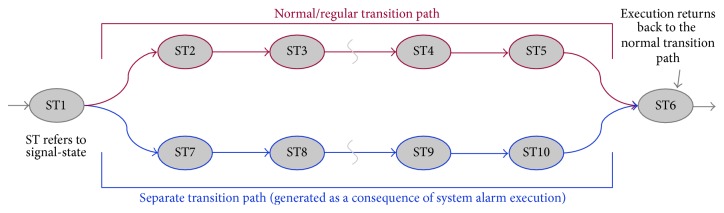
The consequence of system alarm execution.

From the above discussion, it is clear that PLAT is completely automated, does not require much domain knowledge, and can operate within a small memory footprint [space complexity is *O*(*l*(*N* + *M*)), where *l*(≥1) is a small number (depends on the manufacturing system), *N* is total number of PLC I/O signals, and *M* is total number of devices]. So, it can easily handle a large manufacturing system with a reasonable computer configuration. It is easy to realize that if the control process model is fully converged then PLAT does not produce any false positives during fault searching. The model convergence problem can largely be avoided if the model is built on a large reference database. However, sometimes the control process model cannot converge completely because of the system alarms. The system alarms are used for handling irregular situations in a manufacturing system (e.g., an overheated motor causes starting of the coolant circulation). As this type of situation does not occur frequently, its corresponding transition path may not appear in the reference database. PLAT classifies such missing “true” transitions as the invalid transitions (or faults). When operations associated with a system alarm are executed, a separate transition path gets generated in the signal-state I/O model of its corresponding device. However, the execution returns back to the normal transition path after some time (see Figure 12). For this reason, PLAT does not halt its execution immediately after identifying a fault. If the device operation does not return back to the normal transition path in between some user-defined time then PLAT decisively concludes that fault has taken place in the control process (this also helps to avoid several unimportant fault detections). Otherwise, PLAT reports that transition path to the system operator. If the system operator confirms it as a correct transition path then PLAT incorporates it into the signal-state I/O model of its corresponding device. So, the control process model building procedure of PLAT is not a one-time task, but rather an ongoing procedure. In the next section, we provide a detailed empirical evaluation of PLAT.

## 3. Experimental Study, Results, and Discussion

We have performed our experiments on both simulated and real-world databases (all the databases are obtained from UDMTEK Co., Ltd. [45]). All of our algorithms were implemented in *C*++ and compiled using *g*++  4.7.3 (no special optimization flags were used).

### 3.1. Experiments with Small Simulated Databases

**Figure 13:**
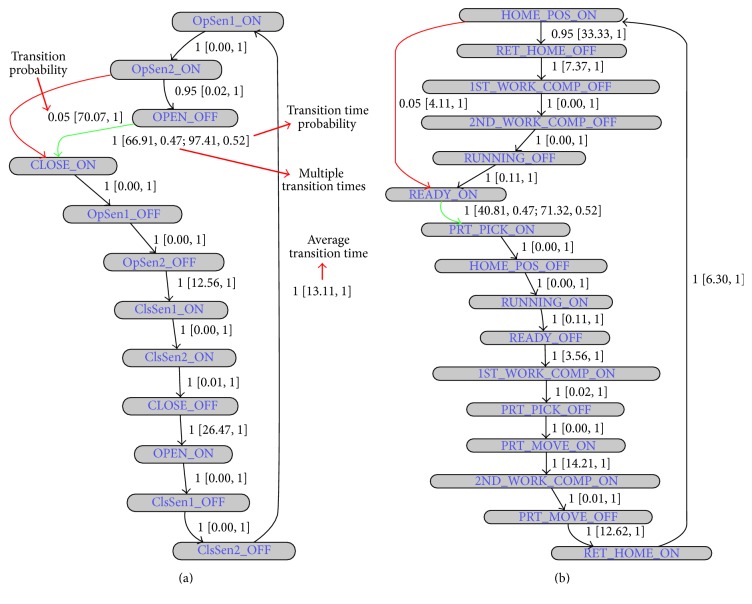
Signal-state I/O models. (a) Signal-state I/O model of Daecha Clamp. (b) Signal-state I/O model of Robot 1.

**Figure 14:**
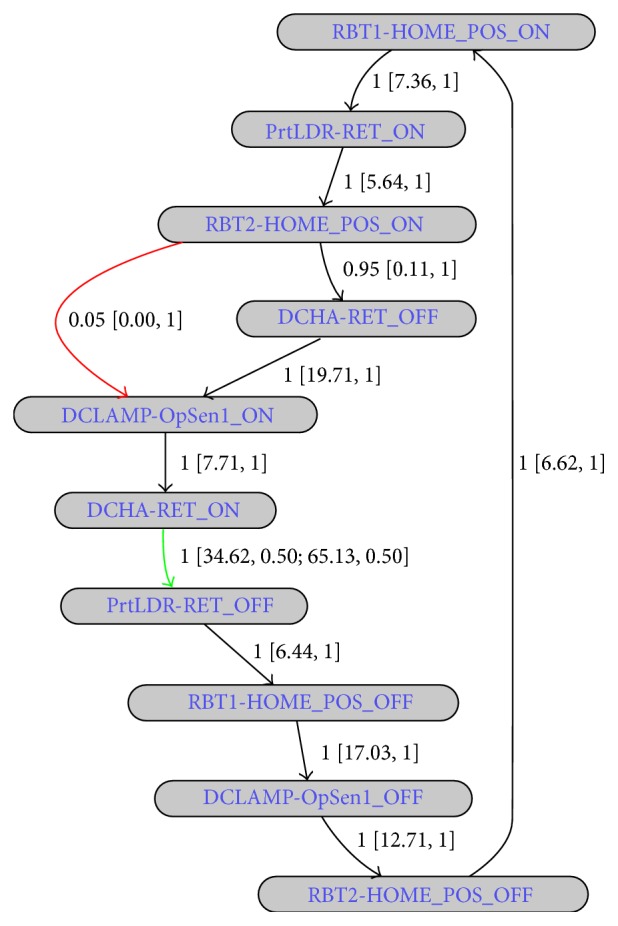
Signal-state I/O model of the group.

We have tested PLAT for various small simulated (or virtual) manufacturing systems in order to find its effectiveness (the computer software simulation is done by using PLC Studio software [46]). Among them, one small scenario is presented in Figure 2. It works as follows: (i) a part is loaded to the Part Loader and then it moves towards Robot 1 through rail track; (ii) after reaching the end position, Robot 1 picks up the part (first task) and then loads it to the Daecha (second task); (iii) Daecha Clamp grips the part and Part Loader returns back to its home position; (iv) after the Daecha Clamp is closed, Robot 2 performs the sealing operation; (v) after the sealing operation is completed, Daecha moves towards its advanced position; (vi) when the Daecha is in its advanced position, Daecha Clamp opens and the part is removed from the system; (vii) then the Daecha returns back to its home position; (viii) this group cycle starts again when another part is set on the Part Loader (a simulation video of this virtual system is provided in [47]). The signal-state I/O models of the Daecha Clamp (called “DCLAMP”), Robot 1 (called “RBT1”), and group found by PLAT are presented in Figures 13(a), 13(b), and 14, respectively. The objectives of the corresponding control signals are given in Table 3 [in those figures, Part Loader, Daecha, and Robot 2 are called “PrtLDR,” “DCHA,” and “RBT2,” resp.]. For the interest of space, signal-state I/O models of the Daecha and Robot 2 are not given (they are similar to the signal-state I/O models of the Daecha Clamp and Robot 1, resp.). In Figures 13 and 14, transition properties are shown in TP  [ATT, TTP; ATT, TTP] format where TP, ATT, and TTP refer to the transition probability, average transition time (given in seconds), and transition time probability, respectively. The models are generated based on a simulated database that contains log data records of the twenty consecutive runs of the system (actually, this is a virtual system and, hence, the state transitions occur very quickly and the model convergence takes place within a very few iterations). Some transitions in those figures have multiple transition times (shown in green color). This is because we presented the parts to the system with interval of (approximately) 30 and 60 seconds (ten times each). The transition time clusters are discarded if the support of a cluster is less than 0.10 (as we have used very few training records), and the clusters are merged if their centers has distance less than 2 seconds. It is easy to see from Figures 13 and 14 that these models give an easy understandable graphical representation of the control process behaviour to operators. Moreover, detailed effects of the faults can easily be found from these representations. We have thoroughly tested the fault detection accuracy of PLAT by making several control signals (given in Table 3) faulty. In all cases, PLAT is able to find accurately all the faults present in the system in real time. The detection results of some of those faults (related to the devices Part Loader, Daecha Clamp, and Robot 1) are given in Table 4. An example fault detection scenario from this experiment is shown in [48], where we tested PLAT by making PRT_CHK signal of the Part Loader device faulty (also see Table 4, first row). As can be seen, PLAT can detect the fault in real time before it propagates through the system [a few examples of the inserted behavioural anomalies and their corresponding detection results are also presented in Table 4. However, they are given for exemplification purposes only (actually, the transition execution or the timing behaviour of a real-world manufacturing system is not generally that very simple)].

### 3.2. Experiments with Real-World Large Databases

We ran this experiment on a desktop computer with 3.20 GHz Intel *i*5 quad-cores CPU and 4 GB of DDR3 main memory, running 64-bit Ubuntu (version 13.04) as the operating system. The processor has 6 MB of *L*3 cache shared by all of its cores. In addition, each core has a private 32 KB *L*1 instruction and *L*1 data cache and 256 KB of *L*2 cache. We collected a log database from a body-in-white (BIW) automotive manufacturing system (controlled by five PLCs) with approximately 4.32 million records for our experiments (among those, approximately 3.32 million log data records were used for model building and 1 million log data records were used for searching faults and behavioural anomalies). The log database was created by taking the log data records from 21,960 PLC signals that operate a total of 1,620 devices (divided into six groups). PLAT found in total 50,567 signal-state transition rules while building the signal-state I/O models for the devices and the groups. The parameter values set by us were as follows: (i) the load factor of the hash tables was set to 0.6 (specifically, the closest prime number to that value); (ii) for the CS*k*-means algorithm, we set the number of clusters value *k* to 5 (in our case, transitions can have maximum of two transition times); (iii) for the HAC algorithm, clusters could only merge if their centers have distance less than 3 seconds; (iv) clusters are discarded if the support of a cluster is less than 0.08 (see Algorithms 1, 2, and 3 for details). The above defined parameter values are domain dependent but can easily be set with a little domain knowledge and using some empirical evaluations.

**Figure 15:**
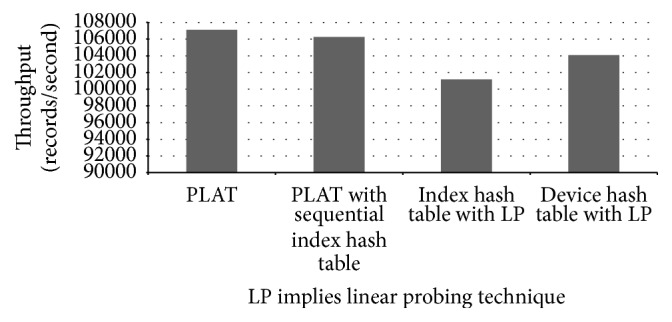
Comparison of throughputs.

**Figure 16:**
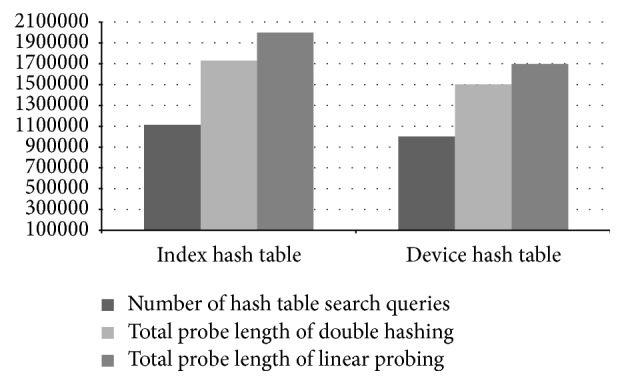
Comparison of probe lengths.

Although we used a high-configuration computer for our experiments, our hash tables actually take in total less than 20 MB of memory. In the control process model building phase, PLAT is able to process 31,527 records per second. Performance of PLAT for various hash table settings is shown in Figure 15. As can be seen in that figure, PLAT can process 107,128 records per second during query database processing phase. Even though computers used in industries have lower processing speed, this is a fairly high value compared to log data records produced per second in the real-world manufacturing industry. So, PLAT can easily detect the control process faults (and transition time anomalies) in real time. This was possible only because of the design choices (such as device-group based process modelling and hash table based model indexing) taken throughout this paper (see Sections 2.3 and 2.5 for details). As can be seen in Figure 15, if we replace the index hash table with the sequential index hash table (see Section 2.5) then it decreases the throughput by 895 records per second. We have observed that this reduction is resulted because of 5.13% increment in the number of cache misses. In the literature, because of this caching effect, it is often argued that, in practice, linear probing is a better choice than double hashing. However, in our case linear probing cannot increase performance by caching mainly because of the large hash table record length. Please note that the (default) size of the index hash table records is 168 bit (see Figure 10) and, hence, a very few records can actually be fit into a single cache line (even in a high-configuration computer). So, linear probing cannot take advantage of more cache hits that could compensate the performance penalty caused by a high number of probe sequences (the same is also true for other hash tables). Recall that most of the time, linear probing produces much longer probe sequences than double hashing (see, e.g., [25, 43]). The comparison results of Figures 15 and 16 also give enough support for this conclusion. As can be seen from Figure 16, if we use linear probing strategy in index hash table, it increases the total probe length by 15.68% over double hashing; and, for the device hash table, this increment is 12.87% (which are quite high increments). However, linear probing is primarily motivated to decrease the number of cache misses through better locality of reference. We have observed that, in case of index hash table, it reduces the cache misses by 13.09% over double hashing; and, for device hash table, this reduction is 12.53%. As we can see in Figure 15, if we use linear probing strategy in the index hash table (resp., device hash table), throughput decreases by 5,980 records (resp., 3,045 records) per second (which means quite high performance degradation in both cases). This gives enough evidence that linear probing cannot take advantage of more cache hits that can compensate the performance penalty caused by high probe sequences. So, in our case double hashing is indeed a better choice than linear probing (as argued previously); and all the design choices taken during the model indexing phase (see Section 2.5 for details) are certainly appropriate. We should mention that similar performance results are also obtained for other manufacturing systems (the results from two additional experiments are shown in the Appendix).

**Figure 17:**
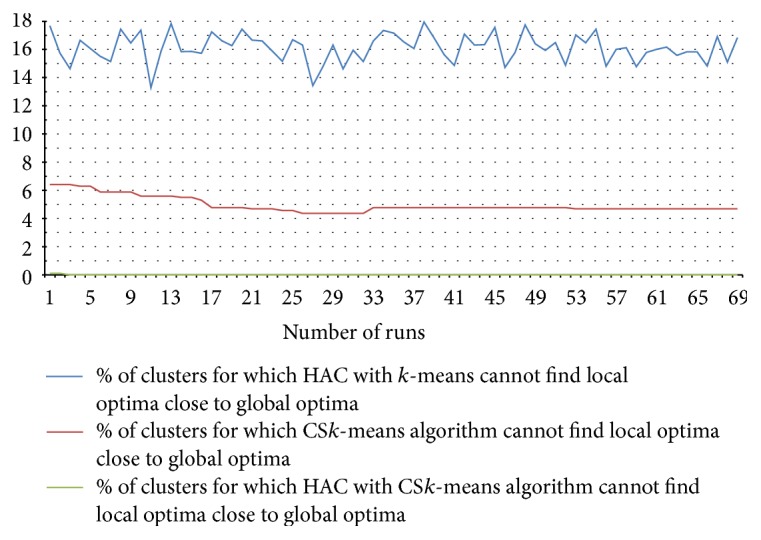
Comparison results of clustering algorithms.

Performance results of the clustering algorithms are given in Figure 17. The result is taken on 7,027 transitions of different devices and groups with a total of (approximately) 0.48 million transition time instances (some of these transitions actually have single transition time, however, with some anomalies). To give an equal platform for comparison, we took data points (transition times) from the sorted data points following the uniform distribution and passed them as seed values to the (traditional) *k*-means algorithm to give the seeds better distribution. In Figure 17, the number of runs specifies the value of* parameter* *ε* in CS*k*-means algorithm (see Algorithms 1 and 2 for details) and the term “local optima close to global optima” refers the local optima within distance of 0.6 seconds to global optima. Actually, for a large percentage of clusters, both CS*k*-means algorithm (>62%) and *k*-means algorithm were unable to find global optima. However, the percentage of clusters for which local optima close to global optima is not found, is approximately 74% for *k*-means (not shown for the interest of space) and for CS*k*-means algorithm, this reaches less than 4% for some suitable values of* parameter  ε* (for *ε* > 16, see Figure 17). The particular importance of our seed value distribution technique lies here. Although, in our CS*k*-means algorithm, we are not able to find global optima for a large fraction of the time, we are able to bind the local optima very close to it most of the time. Most of our transitions (around 65%) take more than 4 minutes to execute and, therefore, the definition of “close” (less than distance of 0.6 seconds) is in practice a very strict bound. As can be seen from Figure 17, when HAC is applied after CS*k*-means algorithm (restricted to two clusters; also see Algorithms 1 and 3), the percentage of clusters for which the local optima close to the global optima is not found is further reduced and reaches zero for some values of *ε* > 2. Actually, we have set our seeding technique in such a way that the clusters are built based on data points that are “far away” from each other (see Section 2.4.2 for details). In some situation where the clusters are not well separated, CS*k*-means algorithm gets trapped in local optima. However, it recovers in the subsequent run of HAC as those clusters are merged (this becomes possible only because CS*k*-means algorithm does not get trapped into poor local optima); and our overall clustering algorithm of Algorithm 1 finds the global optima or local optima that are very close to the global optima. In case of HAC with *k*-means algorithm, the percentage of clusters for which local optima close to global optima is not found is around 16% (which is quite high; see Figure 17). Moreover, most of them are actually very poor local optima (even if we relax the definition of “close” to “less than distance four,” the graph in Figure 17 will not change much). This is because, in *k*-means algorithm, the seed points are simply distributed over the range of data points, and (unlike CS*k*-means algorithm) the distribution of the data points or the distances between the seeds have not been taken into consideration. For this reason, CS*k*-means algorithm always outperforms *k*-means algorithm (note that the result of Figure 17 is taken based on a large number of transitions with varied actual transition times; also see the appendix to get more details about the performance results of the clustering algorithms). In Figure 17, the* parameter  ε* does not have much effect on clustering quality (as stated earlier), if it is not set too low (which means very less penalization of lower-order numbers). If the value is not set too low (*ε* < 3), then our overall clustering algorithm finds solutions within a distance of 0.6 seconds to the global optima in all the cases (which is quite strict bound). Recall from Section 2.4.2 that our goal for clustering is to choose a representative data point and use it to perform classification of the time instances (finding global optima is not really our concern). It is true that if the solutions are far away from global optima, then it can severely affect our transition time error classification scheme. However, as can be seen from Figure 17, our overall clustering algorithm finds solutions very close to the global optima for all the transition times and, hence, does not create an issue for our purpose. If we use a very low value of *k* in CS*k*-means algorithm, then our overall clustering algorithm can end up with some solutions not close to the global optima (in some cases, “true” clusters can be lost due to outliers). However, if we use a slightly higher value of *k* (as done in the above case), then these issues are significantly mitigated and, hence, a slightly higher value of *k* is recommended (we strongly encourage the readers to take a look at the appendix for more information).

The accuracy of identifying transition time errors (or any other types of behavioural anomalies) is hard to determine as it varies depending on system operator's perspective. The operators often set various ranges to identify how compactly the system is working (mostly performed offline). Anyhow, we have tested PLAT on a synthetic database (with the same set of signals and devices as stated above) in order to find this in the laboratory environment. The database was created by inserting transition time errors (1,500 errors inserted, normally distributed within range of 1.0 to 2.5 seconds of “true” maximum transition times) randomly into the original database [we have considered the maximum value of an actual transition time cluster as the true maximum transition time and simply assumed that a transition time error occurs if any transition take 1 second more than its corresponding maximum transition time]. The same experiment is also conducted for faults (150 software-simulated faults are inserted into the database). The fault and transition time error detection accuracy of PLAT is given in Table 5 (averaged over five runs). As can be seen in that table, PLAT detects all the faults present in the system accurately (actually, no faults can remain unidentified in PLAT, as argued in Section 2.3); and, in most of the cases (86% cases), PLAT is able to identify the transition time error correctly (also see the appendix). It is unable to find the remaining transition time errors mainly because of merging of the “actual” transition time clusters. As an example, consider the working procedure of stacker device (a simulation video of the stacker device (similar to the real one) is given in [49]). The stacker device works as follows: (i) the stacker robot moves towards the left or right direction and fixes the stacker clamp in an appropriate position; thus the stacker arm can pick the right part from its storage location; (ii) the stacker arm picks the part and places it on the shuttle; (iii) then the stacker returns back to its home position. As can be seen in [49], signal-state I/O model of the stacker device has multiple transition paths from its starting signal-state node; and, for that reason, some of its transitions actually have multiple transition times, depending on which part the stacker device is processing. However, as they do not vary much (<1 second, on average), the actual transition time clusters are merged. For this reason, in case of stacker device, PLAT is unable to locate some of the transition time errors (the same occurs for the corresponding transition time probabilities). It is hard to eliminate this kind of inaccuracy for an automated tool without any domain knowledge. However, even in this kind of situation, if the transition time errors vary much (3.2 seconds for the above experiment), PLAT can detect all the transition time errors accurately [similar detection accuracy is also achieved in case of the transition and transition time probability errors; however, for the interest of space, we have omitted those redundant results].

We have not compared PLAT with any other approaches mentioned in Section 1 because, they are not intended to identify the faults in real time from a large database; and forcing them to achieve so will be highly unfair. In addition, there is no straightforward way to accomplish that (see Sections 1 and 2.3 for details).

## 4. Conclusion

In this paper, we presented an automated tool called PLAT that can detect faults and behavioural anomalies associated with PLC control processes in real time. Our experimental section shows that PLAT is significantly fast, can process more than 100 K log data records per second, and can easily execute within a small memory footprint. From the structures of hash tables and the working procedure of PLAT, it is easy to perceive that the fault and behavioural anomaly detection procedure of PLAT has linear space and constant time complexity. Our experiments show that PLAT can easily handle a large manufacturing system with a reasonable computer configuration and can be installed in parallel to the data logging system in order to identify faults and behavioural anomalies instantly. PLAT can accurately identify all the faults present in the PLC signal log database. Some less significant behavioural anomalies can remain unidentified; however, the number of such anomalies will not be so high in practice. PLAT can also handle informationally centralized manufacturing systems efficiently. This becomes possible because of our top-down hierarchical (or group-device based) control process model structure. Future work planned will be to

- refine the algorithms of PLAT so that it can identify the root causes of the faults and behavioural anomalies and can suggest corrective actions;
- investigate models with predictive and preventive fault finding features.

## Figures and Tables

**Figure 18:**
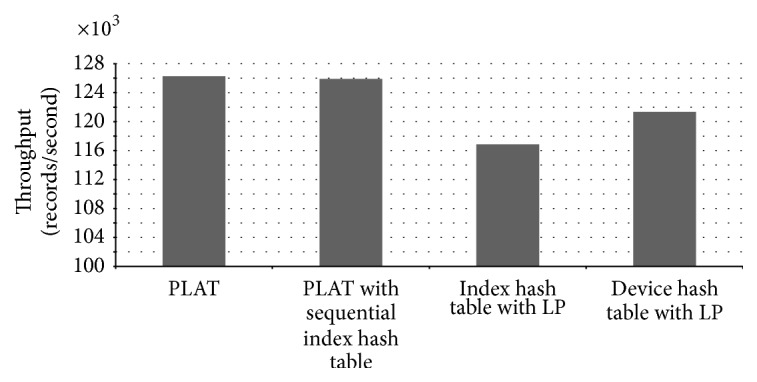
Comparison of throughputs for Database A.

**Figure 19 fig19:**
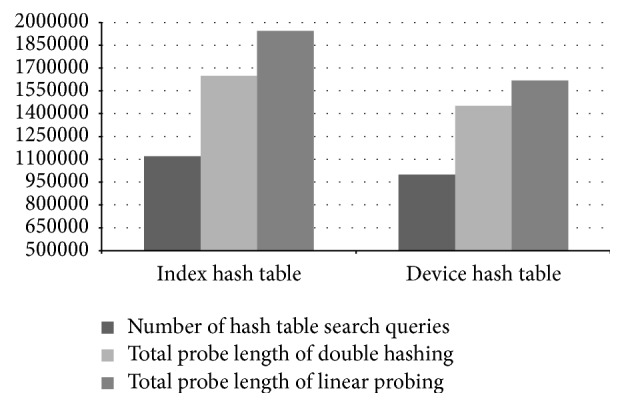
Comparison of probe lengths for Database A.

**Figure 20 fig20:**
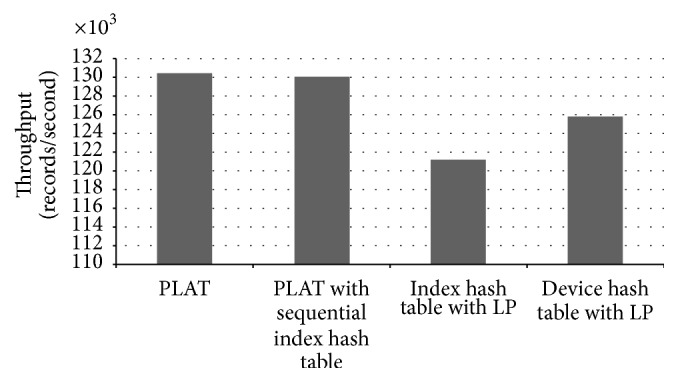
Comparison of throughputs for Database B.

**Figure 21:**
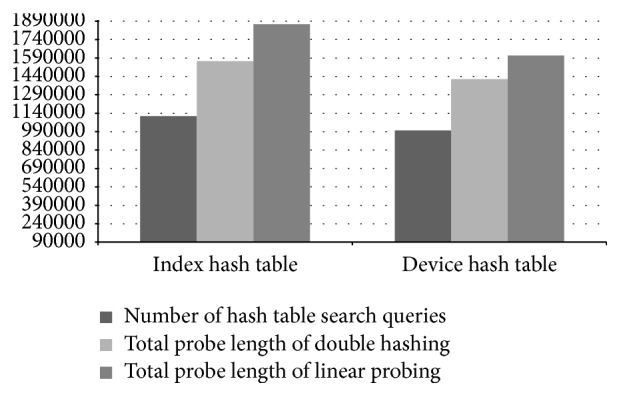
Comparison of probe lengths for Database B.

**Figure 22:**
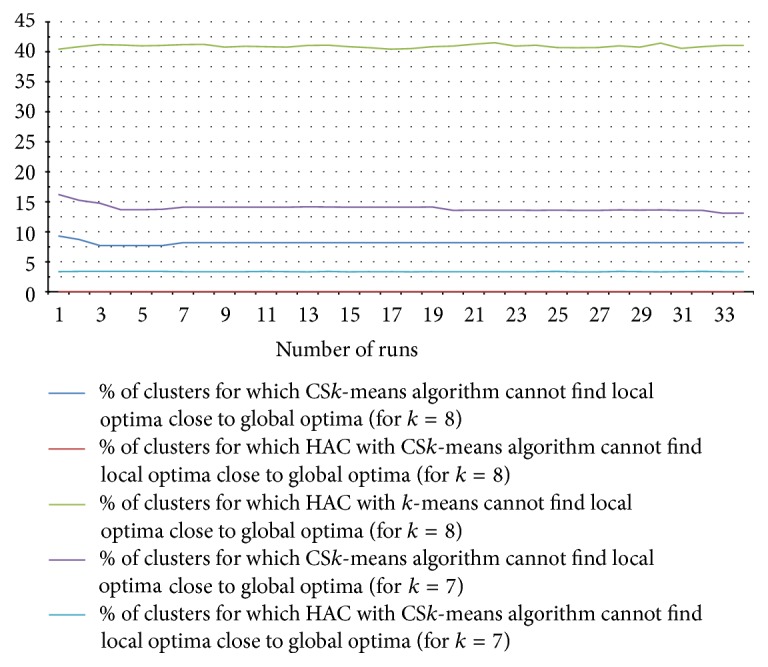
Comparison results of clustering algorithms for Database A.

**Figure 23:**
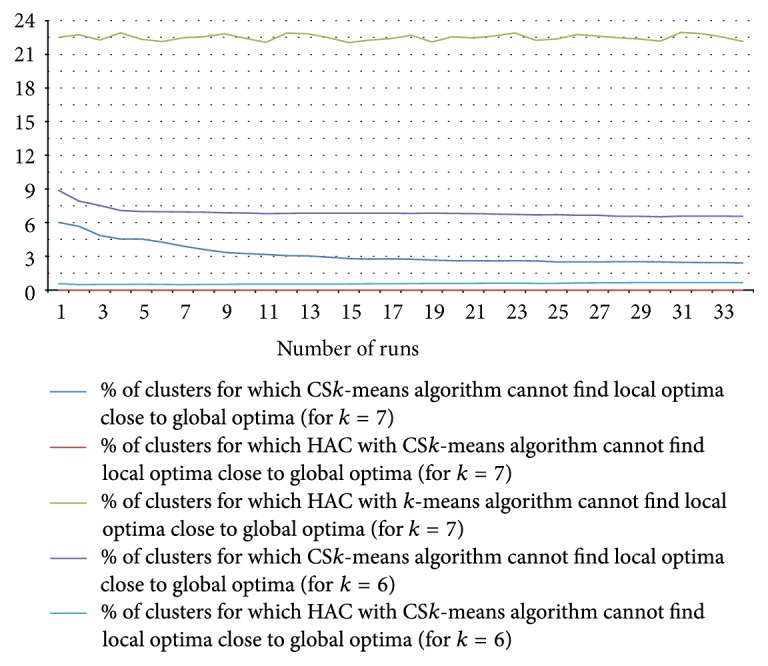
Comparison results of clustering algorithms for Database B.

**Algorithm 1:**
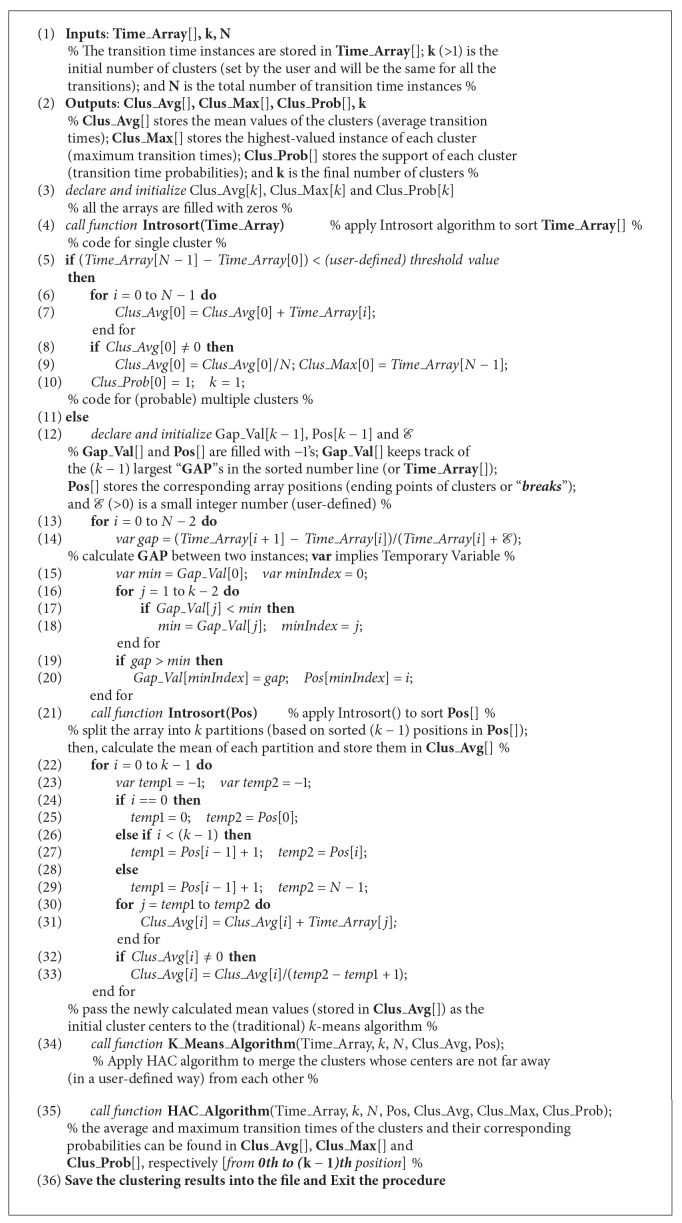
Transition time clustering algorithm (CS*k*-means algorithm with HAC).

**Algorithm 2:**
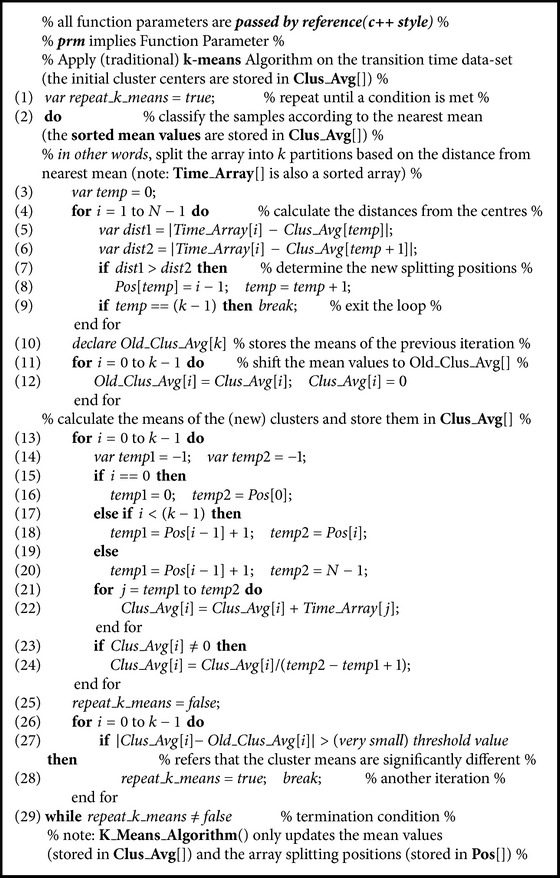
**Function** **K** **_Means_Algorithm**().

**Algorithm 3:**
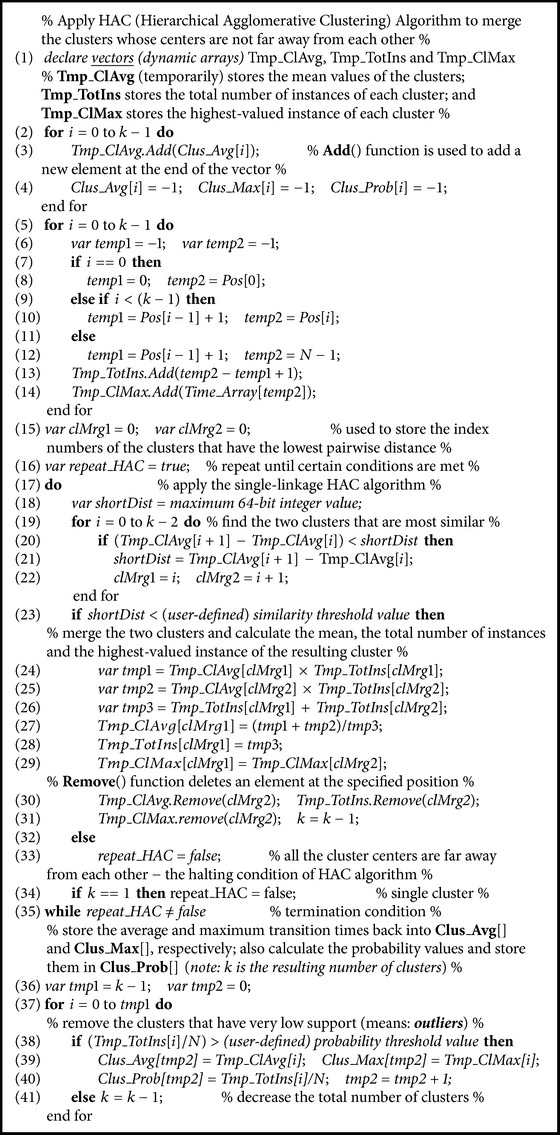
**Function HAC_Algorithm**().

**Table 1:** Log data format.

Time stamp	Symbol name	Status
14:45:34.17	Group2-Device1-Signal2	1
14:46:57.12	Group1-Device3-Signal1	1
14:48:14.31	Group2-Device1–Signa12	0
14:49:19.23	Group1-Device1-Signal1	1
14:52:41.15	Group1-Device1-Signal3	1

**Table 2:** Example set of transition rules.

Current signal state	Next signal state	(Example) transition probability	(Example) average transition time		(Example) maximum transition time		(Example) transition time probability	
Group1-Device2-Signal2_OFF	Group1-Device2-Signal2_ON	0.7	20.34		20.35		1	
Group1-Device2-Signal2_OFF	Group1-Device2-Signal4_ON	0.3	51.08		51.11		1	
Group1-Device2-Signal2_ON	Group1-Device2-Signal4_ON	1	15.07		15.13		1	
Group1-Device2-Signal4_OFF	Group1-Device2-Signal2_OFF	1	8.44		8.45		1	
Group1-Device2-Signal4_ON	Group1-Device2-Signal4_OFF	1	20.45^*∗*^	60.02^*∗*^	20.51^*∗*^	60.15^*∗*^	0.3^*∗*^	0.7^*∗*^

^*∗*^Two transition times and their corresponding transition time probabilities.

**Table 3:** Description of signals: objectives of the control signals.

	Signal name	Type	Description
DCLAMP	OpSen1	Sensor input	Identifies if left and right clamps are opened
	OpSen2	Sensor input	
	CLOSE	Output	Notifies clamp closing operation
	ClsSen1	Sensor input	Identifies if left and right clamps are closed
	ClsSen2	Sensor input	
	OPEN	Output	Notifies clamp opening operation

RBT1	HOME_POS	Sensor input	Identifies if robot is in its home position
	READY	Output	Notifies robot is ready to start its work
	PRT_PICK	Output	Notifies robot is picking up the part from the Part Loader
	RUNNING	Sensor input	Identifies if robot is in running mode
	1ST_WORK_COMP	Sensor input	Identifies if robot picked up the part from the Part Loader (work completion signal)
	PRT_MOVE	Output	Robot is moving the part of Daecha
	2ND_WORK_COMP	Sensor input	Identifies if robot placed the part on Daecha (work completion signal)
	RET_HOME	Output	Notifies robot is returning to its home position (will be ON until part from Daecha is removed)

Group	RBT1-HOME_POS	Starting signal of Robot 1	Sensor signal that identifies if robot RBT1 is in its home position
	PrtLDR-RET	Starting signal of Part Loader	Sensor signal that identifies if Part Loader PrtLDR is in its home position
	RBT2-HOME_POS	Starting signal of Robot 2	Sensor signal that identifies if robot RBT2 is in its home position
	DCLAMP-OpSen1	Starting signal of Daecha Clamp	Sensor signal that identifies if left clamp of Deacha Clamp DCLAMP is opened
	DCHA-RET	Starting signal of Daecha	Sensor signal that identifies if Daecha DCHA is in its home position

**Table 4:** Fault and behavioural anomaly detection result.

PLAT tool	Fault or behavioural anomaly description	Faulty or erroneous transition	Expected transition/s	Affected device
1	The sensor signal PRT_CHK of the Part Loader (PrtLDR) is turned ON while the Part Loader (PrtLDR) is returning back to its home position	PrtLDR-ADV_OFF → PrtLDR-PRT_CHK_ON	PrtLDR-ADV_OFF → PrtLDR-RET_ON	Part Loader (PrtLDR)

2	The sensor signal OpSen1 of the Daecha Clamp (DCLAMP) is permanently^*∗*^ turned ON while Robot 1 (RBT1) is picking up the part from the Part Loader (PrtLDR) [^*∗*^the sensor input will always be 1 even if the left clamp is not opened]	DCLAMP-CLOSE_ON → DCLAMP-OpSen2_OFF	DCLAMP-CLOSE_ON → DCLAMP-OpSen1_OFF	Daecha Clamp (DCLAMP)

3	The actuator related to the signal OPEN of the Daecha Clamp (DCLAMP) is decelerated while the clamps are being opened	DCLAMP-OPEN_ON → DCLAMP-ClsSenl_OFF	Not applicable [transition time error has occurred] Expected (maximum) transition time: 0.03 and observed transition time: 2.11 (in seconds)	Daecha Clamp (DCLAMP)

4	The sensor signal 1ST_WORK_COMP of Robot 1 (RBT1) is turned OFF while Robot 1 (RBT1) is transferring the part to the Daecha (DCHA)	RBT1-PRT_MOVE_ON → RBT1-1ST_WORK_COMP_OFF	RBT 1-PRT_MOVE_ON → RBT1-2ND_WORK_COMP_ON	Robot 1 (RBT1)

5	The actuator related to the signal FORWARD of the Part Loader (PrtLDR) is deactivated^*∗*^ while the part is being loaded to the Part Loader (PrtLDR) [^*∗*^if an actuator is deactivated then the corresponding mechanical operation cannot be carried out]	The subsystem has stopped working [PLAT has already waited for 180 seconds]	Last observed signal state: PrtLDR-FORWARD_ON	Part Loader (PrtLDR)

6	Seven parts are presented to the system with interval of (approximately) thirty seconds and twenty-three parts are presented to the system with interval of (approximately) sixty seconds [the periodic checking of the probability values is done after every thirty group cycles]	PrtLDR-BACKWARD_OFF → PrtLDR-PRT_CHK_ON	Not applicable [transition time probability error has occurred] Expected probabilities: 0.47 and 0.52 and observed probabilities: 0.20 and 0.79	Part Loader (PrtLDR)

7	The sensor signal RET of the Part Loader (PrtLDR) is turned OFF while the Part Loader (PrtLDR) is in its home position [during the first group cycle]	PrtLDR-RET_ON → PrtLDR-RET_OFF	PrtLDR-RET_ON → PrtLDR-BACKWARD_OFF OR PrtLDR-RET_ON → PrtLDR-PRT_CHK_ON	Part Loader (PrtLDR)

8	The sensor signal HOME_POS of Robot 1 (RBT1) is turned ON while Robot 1 (RBT1) is placing the part to the Daecha (DCHA)	RBT1-2ND_WORK_COMP_ON → RBT1-HOME_POS_ON	RBT1-2ND_WORK_COMP_ON → RBT1-PRT_MOVE_OFF	Robot 1 (RBT1)

9	The actuator related to the signal BACKWARD of the Part Loader (PrtLDR) is decelerated while the Part Loader (PrtLDR) is returning back to its home position	PrtLDR-BACKWARD_ON → PrtLDR-ADV_OFF	Not applicable [transition time error has occurred] Expected (maximum) transition time: 0.04; and observed transition time: 2.57 (in seconds)	Part Loader (PrtLDR)

10	The sensor signal ClsSenl of the Daecha Clamp (DCLAMP) is deactivated^*∗*^ while the Part Loader (PrtLDR) is proceeding towards its advanced position [^*∗*^if a sensor is deactivated then it is not able to send any further input value to the PLC controller]	DCLAMP-OpSen2_OFF → DCLAMP-ClsSen2_ON	DCLAMP-OpSen2_OFF → DCLAMP-ClsSenl_ON	Daecha Clamp (DCLAMP)

11	The actuator related to the signal PRT_PICK of Robot 1 (RBT1) is deactivated while Daecha (DCHA) is returning back to its home position	The subsystem has stopped working [PLAT has waited for 180 seconds]	Last observed signal state: RBT1-PRT_PICK_ON	Robot 1 (RBT1)

12	The actuator related to the signal PRT_MOVE of Robot 1 (RBT1) is decelerated while Robot 1 (RBT1) is loading the part to the Daecha (DCHA)	RBT 1-PRT_MOVE_ON → RBT1-2ND_WORK_COMP_ON	Not applicable [transition time error has occurred] Expected (maximum) transition time: 16.01; and observed transition time: 24.56 (in seconds)	Robot 1 (RBT1)

In this table, the arrow symbol (→) represents the signal-state transition; and rows numbers 3, 6, 9, and 12 are the examples of behavioural anomalies [others are the examples of faults].

A transition time error occurs if any transition takes longer time than the following: (its corresponding maximum transition time + user-defined threshold value Δ).

Δ = 1.5 if Max_Time ≤ 15; Δ = 3 if Max_Time > 15 and ≤ 30; Δ = 4 if Max_Time > 30 [Max Time: maximum transition time; all the times are given in seconds].

A transition time probability error occurs if significant changes (i.e., the changes greater than 0.15) in the transition time probabilities associated with a transition take place.

The name of the “Affected Device” can easily be determined from the name of signal-states associated with the faulty/erroneous transition (this column is continually updated based on the propagation of the fault/behavioural anomaly).

**Table 5:** Accuracy of the fault and behavioural anomaly detection procedure of PLAT.

Total number of inserted faults	Total number of identified faults	Accuracy of fault identification	Total number of inserted transition time errors	Total number of identified transition time errors	Accuracy of transition time error identification
150	150	100%	1500	1304	86.93%

**Table 6:** Accuracy of the fault and behavioural anomaly detection procedure of PLAT.

	Total number of inserted faults	Total number of identified faults	Accuracy of fault identification	Total number of inserted transition time errors	Total number of identified transition time errors	Accuracy of transition time error identification
Database A	150	150	100%	500	461	92.20%
Database B	150	150	100%	500	449	89.80%

## Appendix

### Experiments with Two Additional Real-World Large Databases

We acquired two log databases from two different BIW automotive manufacturing systems for our experiments. Brief details of those two databases are given in the following:

(i) The first database (called Database A) contains approximately 3.70 million log data records. Among those, approximately 2.70 million log data records were used for model building and 1 million log data records were used for searching faults and behavioural anomalies. The log database was created by taking the log data records from 14,467 PLC signals that operate a total of 1,127 devices (divided into four groups).
(ii) The second database (called Database B) contains approximately 3.45 million log data records. Among those, approximately 2.45 million log data records were used for model building and 1 million log data records were used for searching faults and behavioural anomalies. This log database was created by taking the log data records from 13,896 PLC signals that operate a total of 973 devices (divided into four groups).

While building the signal-state I/O models for the devices and groups, PLAT found in total 33,394 and 31,976 signal-state transition rules for Database A and Database B, respectively [all the experimental settings are the same as specified in Section 3.2 (1st paragraph)]. In both cases, PLAT takes in total less than 18 MB of memory. For Database A, the performance result of PLAT for various hash table settings is shown in Figure 18 and the comparison result of the probe lengths (linear probing versus double hashing technique) is given in Figure 19. For Database B, the performance result of PLAT for various hash table settings is given in Figure 20 and the comparison result of the probe lengths is presented in Figure 21. From the experimental results presented in Figures 18 [Fig fig19] [Fig fig20]–21, we can draw the following conclusions:

(i) PLAT is significantly fast; it can process more than 125 K log data records per second (which means PLAT can effectively detect the control process faults in real time).
(ii) In our case, the double hashing probing technique always exhibits a much better performance compared to the linear probing technique. If we use linear probing strategy in our hash tables, it increases the probe length up to 19.31 percent and decreases the throughput up to 9,424 records per second (which is quite high decrement).
(iii) If we replace the index hash table with the sequential index hash table, it always decreases the throughput of PLAT (not very much; it decreases the throughput up to 474 records per second).
(iv) So, all the design choices taken during the hash table structure and the control process model designing phase (see Sections 2.3 and 2.5 for details) indeed improve the performance of PLAT.

The rationale behind the above results is thoroughly explained in Section 3.2 (2nd paragraph).

Performance results of the clustering algorithms for Database A and Database B are given in Figures 22 and 23, respectively. For Database A, the performance result was taken on 4,976 transitions with in total (approximately) 0.44 million transition time instances; and, for Database B, the performance result was taken on 3,383 transitions with in total (approximately) 0.28 million transition time instances. The transitions can have maximum of three transition times [all the other clustering parameter values and the experimental settings are the same as specified in Section 3.2 (1st and 3rd paragraph)]. To summarize the presented results, the following conclusions can be drawn:

(i) HAC with CS*k*-means algorithm always outperforms HAC with *k*-means algorithm.
(ii) If we set the number of clusters values *k* to 8, in very few cases, CS*k*-means algorithm is not able to find the local optima close to global optima (on average approximately 8% and 3% of cases for Database A and Database B, resp.). When HAC is applied after CS*k*-means algorithm, the percentage of clusters for which the local optima close to global optima is not found is further reduced and reaches zero for both databases. So, our clustering algorithm finds solutions very close to the global optima for all the cases and, hence, does not create an issue for our error classification purpose.
(iii) If we set the number of clusters values *k* to 7 (lower value of *k*), in relatively large number of cases, CS*k*-means algorithm is not able find the local optima close to global optima (on average approximately 14% and 7% of cases for Database A and Database B, resp.). Moreover, the clustering algorithm of Algorithm 1 ends up with some solutions not close to the global optima (on average approximately 3% and 0.6% of cases for Database A and Database B, resp.). This is the reason why a slightly higher value of *k* is recommended.
(iv) The* parameter  ε* does not have much effect on clustering quality if it is not set too low (we recommend to set the value of* parameter  ε* close to 20, as it always exhibits a quite good performance result).
(v) We have observed that the average number of iterations of CS*k*-means algorithm (2.17 iterations, on average) is much lower than *k*-means algorithm (4.89 iterations, on average).

The definition of “local optima close to global optima” and* parameter ε* can be found in Section 3.2 (3rd paragraph) and Algorithm 1, respectively. The rationale behind the above results is thoroughly explained in Section 3.2 (3rd paragraph).

We have tested PLAT on some synthetic databases in order to find its fault and transition time error identification accuracy. The synthetic databases were created by inserting the faults (150 software-simulated faults are inserted) and the transition time errors (500 errors are inserted, normally distributed within range of 1 to 3 seconds of maximum transition times) randomly into the original databases [all the other experimental settings are the same as discussed in Section 3.2 (4th paragraph)]. The results of this experiment are provided in Table 6 (averaged over five runs). Based on the results presented, the following conclusions can be made:

(i) PLAT can accurately identify all the faults present in the PLC signal log database (we have also empirically found that the NDAAO automaton based approaches [10–12] can detect all the faults present in the log database).
(ii) Some less significant transition time errors can remain unidentified; however, the number of such anomalies will not be so high in practice (as can be seen in Table 6, it is less than 11%).

Please note that in all the above cases, if the inserted transition time errors vary much (>3.7 seconds) then PLAT can detect all the transition time errors accurately.
